# The multifactorial approach and the food allergen-specific substitutive diet as a tool to manage and ameliorate adverse reactions to foodstuffs in adulthood: study protocol for a randomized controlled trial—the ALASKA study

**DOI:** 10.1186/s13063-024-08307-2

**Published:** 2024-07-20

**Authors:** Lisset Pantoja-Arévalo, Eva Gesteiro, Margarita Pérez-Ruiz, Jaime López-Seoane, Patricia Wusterhausen, Torsten Matthias, Rafael Urrialde, Marcela González-Gross

**Affiliations:** 1 ImFINE Research Group, Department of Health and Human Performance, Universidad Politécnica de Madrid, 28040 Madrid, Spain; 2 Department of Research and Development, Aesku.Diagnostics GmbH, 55234 Wendelsheim, Germany; 3 Department of Genetics, Physiology and Microbiology, Faculty of Biological Sciences, Universidad Complutense de Madrid, 28040 Madrid, Spain; 4 Department of Pharmaceutical and Health Sciences, Faculty of Pharmacy, Universidad CEU San Pablo, 28003 Madrid, Spain; 5 Department of Nutrition, Faculty of Nursing, Universidad de Valladolid, 47002 Valladolid, Spain; 6 Biomedical Research Center of Pathophysiology of Obesity and Nutrition-CIBERobn, Carlos III Health Institute, 28029 Madrid, Spain

**Keywords:** Clinical trial, Diet, Disease management, Food hypersensitivity, Food intolerance, Nutrition therapy

## Abstract

**Background:**

Adverse reactions to foodstuffs (ARFS), specifically food allergy (FA) and food intolerance (FI), are increasing worldwide and represent a major public health concern. Thus, ARFS management, its identification, evaluation, and intervention, must provide a comprehensive solution.

**Objectives:**

(a) To develop a multifactorial strategy for ARFS management in adults with FA and/or FI; (b) to describe the multiple influential variables in ARFS within the realm of ARFS management; and (c) to design a personalized food allergen-specific substitutive diet (FASSD), as a 6-month dietary treatment option for adults with ARFS and as a component of ARFS management.

**Methods:**

The ALASKA study will consider the following main variables as part of the ARFS management: (1) demographics and clinical information; (2) symptomatology, food and beverages intake and physical activity; (3) hematobiochemical study; (4) immunology; (5) enzymatic activity; (6) anthropometry, body composition, and physical fitness; (7) QoL; (8) 6-month intervention; (9) end of the study; and (10) other assessments. The FASSD will be designed with special emphasis on the commonly lacking micronutrients in the ARFS population: niacin, Mg, K, P, Ca, Zn, B12, folate, Fe, and fiber.

**Discussion:**

The ALASKA study protocol has been developed as a global strategy to manage and evaluate ARFS in Spanish adults older than 18 years of age. Approaching ARFS with multiple assessments, as influencing factors, will lead to a novel strategy for ARFS management. The FASSD has been designed as a personalized tool to avoid crucial micronutrient deficiencies that a current strict food allergen avoidance or elimination diet may provoke.

**Trial registration:**

The protocol has been approved by the Ethics Committee of the UPM (REF.20200602) and registered on ClinicalTrials.gov (NCT05802017).

**Supplementary Information:**

The online version contains supplementary material available at 10.1186/s13063-024-08307-2.

## Introduction

**Fig. 1:**
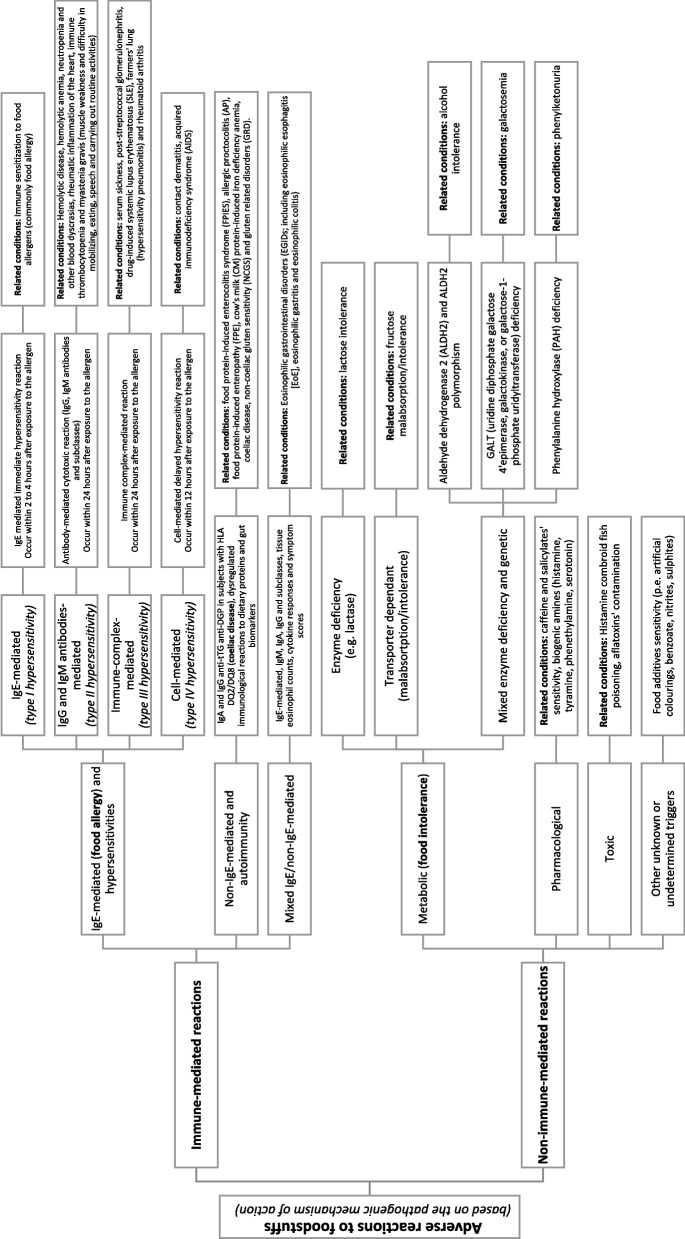
Adverse reactions to foodstuffs classification updated and adapted from a selected literature (described below). Adapted from: Immune-mediated reactions: [6–9]; Non-immune-mediated reactions: [7, 10, 11]

Adverse reactions to foodstuffs (ARFS) are based on an individual’s specific response to food components and have been generally classified by pathogenic mechanism of action and attributed mostly to food allergy (FA) and/or food intolerance (FI) (Fig. 1) [1]. Currently, more than 150 million Europeans suffer from chronic allergic disease, including about 20 million suffering from FA and more than 80 million from FI [2, 3]. Prevalence of ARFS, especially the ones related to FA and FI, has been increasing over the past decades in developed and developing countries becoming an important public health concern [4]. Given that the reactions to foodstuffs are often regarded as pediatric problems, these reactions have not been studied in-depth in adults and no accurate strategies focusing on this population have been established. It has been estimated that by 2050, 50% of the world’s population will be affected by at least one type of allergy; and as one in every five people globally will be aged 60 years or over by that time, the general health and quality of life (QoL) of the adult population emerges as an essential object of study for the coming years [5].

The *gold standard* to diagnose immune-mediated and non-immune-mediated reactions to foodstuffs is the oral food challenge (OFC) [12]. However, to avoid procedures that may imply a prolonged regular practice, there are short breath tests available, such as the Cerascreen® Lactose/Fructose hydrogen H_2_ and methane CH_4_ breath test kit (Cerascreen GmbH, Schwerin, Germany). Similarly, there are clinically relevant blood biomarkers, such as specific immunoglobulins (mostly food-specific IgE antibody (Ab) reactions for type I hypersensitivity or type I food allergy (T1FA)), that together with a clinical history of symptoms can help in diagnosis [13, 14]. In addition, other physical and clinical tests might also contribute to the clarification of the study of specific populations, such as athletes, who may present different physical activity levels (PAL). The presence of ARFS in individuals with particular symptomatology could be linked to gastrointestinal, skin, and/or nervous system (NS) conditions, specifically when having type II hypersensitivity or type II food allergy (T2FA) [15, 16].

In fact, T2FA has been one of the most recently and newly explored hypersensitivities as a tool in the development of ARFS research. Figure 1 outlines the ARFS classification according to the pathogenic mechanism of action. The clinical relevance of food-specific IgG_4_ Ab in food allergy is currently unclear and not recommended to be used alone as a food allergy predictor. It has been observed that IgE/IgG_4_ ratios can be used to enhance the diagnostic and prognostic performance of sIgE [17].

Current treatments of ARFS are not optimal nor ideal from the nutritional and physical condition point of view. For T1FA, treatment consists of strict food allergen avoidance and the use of adrenaline (for allergen-induced systemic reactions), epinephrine, and/or a 24-h dietary recall tool [18]. The application of treatments such as strict food allergen avoidance may cause nutritional deficiencies in certain cases of multiple food allergen responses due to the avoidance of important food groups. For instance, milk avoidance in adults may result in calcium deficiency [19] and lower intakes of zinc and vitamin B2. Likewise, prolonged gluten-free diet make individuals prone to the development of, most notably folate deficiency and lower intakes of calcium, iron, zinc, folate, and fiber; affecting their nutritional status and altering the intestinal microbiota [20]. Substitutive diets suggesting the replacement of allergy-related food for allergen-free food have shown symptoms’ score improvement. Hence, the 6-month intervention of the ALASKA study, to manage and ameliorate ARFS in adulthood, consists in the implementation of a general nutritional advice with dietary recommendations for a healthy lifestyle for subjects in the control group; and a 6-month food allergen-specific substitutive diet (FASSD) for subjects in the intervention group. These effects are greater in subjects with specific pathologies: atopic dermatitis [21], urticaria [22], and gastrointestinal disorders [23].

Consequently, a novel strategy involving the assessment of multiple variables and a FASSD to manage and ameliorate ARFS is proposed in this study. The aim of this study is (a) to develop a multifactorial strategy for ARFS management in adults with FA and/or FI; (b) to describe the multiple influential variables in ARFS within the realm of ARFS management; and (c) to design a personalized FASSD, as a 6-month dietary treatment option for adults with ARFS and as a component of ARFS management.

## Materials and method

### Study design

**Fig. 2:**
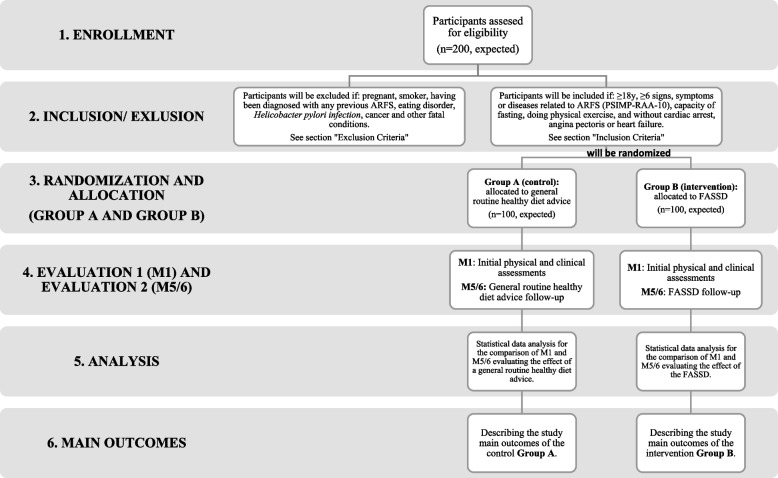
Flow diagram of the ALASKA study. ARFS: adverse reactions to foodstuffs; FASSD: food allergen-specific substitutive diet; M1: physical and clinical assessments at month 1; M5/6: physical and clinical assessments at month 5 or 6; PSIMP-RAA-10: Pathologies and Symptomatology Questionnaire associated with Adverse Reactions to Foodstuffs; y: years of age

This will be a randomized controlled trial (RCT), the Allergies and Food Intolerances in Adults and Athletes study (ALASKA study), with matched pair trial design, exploratory framework, and a 1:2 allocation ratio between the control (Group A) and the intervention group (Group B) respectively, conducted in the Universidad Politécnica de Madrid (UPM) located in the Region of Madrid, Spain. The flow diagram of this study is shown in Fig. 2. The following chart meets The Standard Protocol Items: Recommendations for Interventional Trials (SPIRIT) [24].

### Participants

#### Recruitment

Volunteers with signs, symptoms, and/or diagnosed diseases related to ARFS and older than 18 years, including men and women, will be recruited. The recruitment period will be active for approximately 1 to 3 months and according to the enrollment response. Enrollment mass e-mail campaigns, social media, digital display screens in public areas of the UPM, and the local online contact flyer/website (https://short.upm.es/kwgpo) of the ImFINE Research Group (ImFINE-RG) will be used to recruit interested adults to participate in the ALASKA study. The first approach with possible participants will take place with the use of phone informative conversations between the researcher and the possible participant to share a wider vision about the trial. Engaged participants will be registered in the study and an anonymous participant code will be assigned (see the “ Data storage” section). Participants will initially complete and sign the Informed consent form (Appendix 1), the Inclusion and exclusion criteria form (Appendix 2), the Demographics and clinical characteristics form (Table 3), and the Pathologies and Symptomatology Questionnaire associated with Adverse Reactions to Foodstuffs (PSIMP-RAA-10). After completing these initial questionnaires, participants will be enrolled and their data will be checked for eligibility by a researcher of the ALASKA study. If extra measures are needed to recruit a sufficient number of participants, the recruitment period will be expanded and all the steps of the recruitment phase will be repeated (expected enrollment rate of approximately 150%). Participants will not receive any economic compensation for volunteering in this study, but they will receive personalized nutritional advice and individual reports of all the performed clinical and physical measurements.

The Research Electronic Data Capture Platform (RedCap®), hosted in the Supercomputing and Visualization Center of Madrid Community of the UPM (CESVIMA-UPM) will be used to digitally secure and perform all types of communication, whether massive or individual. RedCap® platform is a specialized tool to build and manage clinical trials and their corresponding databases. All questionnaires and documents (including Appendix 1, Appendix 2, Table 3, the PSIMP-RAA-10 questionnaires, and any other) that will be used during the ALASKA study will be uploaded and will be shared to participants, when applicable, via the RedCap® platform.

#### Sample size and missing data

The required sample size with 95% of power was calculated using the G*Power software v. 3.1 (Heinrich-Heine-Universität Düsseldorf, Düsseldorf, Germany) [25] and previously related-published data [15] and datasets [16]. The cross-sectional study requires a sample size of *n* = 154 (*d* = 0.3, *α* = 0.05). Meanwhile, the longitudinal study requires a sample size of *n* = 33 for the control group and *n* = 65 for the intervention group (*d* = 0.8, *α* = 0.05).

Even if the participants have agreed to join the ALASKA study voluntarily and signed the informed consent form, they will be allowed to abandon the study at any time, without having to provide a specific argument or being penalized (loss to follow-up, missing data, dropout, or similar). Likewise, the researchers of this study reserve the right to end any participation, according to each researcher’s criteria, for the benefit of the research study (e.g., injuries, long trips, low commitment, outbreak of severe diseases, or similar). Due to the nature of the dietary interventions, modifying allocated interventions is not foreseen in this study. However, if a subject chooses to discontinue the intervention on their own or if the researcher decides to stop a participant's intervention for any reason, it will be documented in each participant's individual case report and in the End of Study Form (Annex VIII). Subjects will be able to explain, or not, the reason for voluntarily abandoning the study.

#### Data storage

Participants’ alphanumeric codification will be assigned in RedCap® adopting the format “ALXXXX” (two static letters, pointing the study name, ALASKA study, and four random-digit unique code). Complete records of each participant will be stored in RedCap® platform, locally hosted in the CESVIMA-UPM. Digital databases of each assessment and instrument will be created promoting the data quality and avoiding the need of duplicating questionnaire measurements. Databases will be locked using a secure password and with access to the coordinator and principal investigator (PI) of the ALASKA study (L.P.-A., M.G.-G). Additional researchers, after the authorization of the PI (M.G.-G), will have also access to the password and the study databases. M.G.-G will make final decisions regarding the RedCap® platform management, trial supervision, and the trial start/termination point. RedCap® platform provides the “Data Exports, Reports, and Stats” module, which allows to view reports, inspect plots and descriptive statistics of the data, at any point/time of the trial, as well as export data to Microsoft Excel, SAS, Stata, R, or SPSS for analysis (if access provided by M.G.-G to the researchers of the Alaska study). Complete individual records will be documented using the unique and anonymous participant code and will not contain identifiable participants’ information. Routine day-to-day audits; weekly back-ups of the datasets; and monthly internal audits of the available services (water and electricity) and resources (laboratory equipment) will be performed to support data quality.

Subjects of the study will be selected according to the following described inclusion and exclusion criteria and if they have completed the required preliminary questionnaires: Informed consent model (Appendix 1), Inclusion and exclusion criteria form (Appendix 2), and the Pathologies and Symptomatology Questionnaire associated with Adverse Reactions to Foodstuffs (PSIMP-RAA-10) [14].

#### Inclusion criteria

Enrolled participants who will accomplish the following inclusion criteria will be eligible subjects of study: (1) participants without a pacemaker, prosthetic devices, or metal implants; (2) participants with availability of time to attend two venous blood sample extractions and to dedicate at least 30 min to complete online questionnaires related to this study; (3) participants with the possibility, and not restricted by medical advice, of fasting for at least 8 h (for the extraction of blood samples) and doing physical exercise (including the physical capacity of going up and down stairs); (4) participants that, during physical exercise, have not experimented any chest pain, chest stiffness-tightness, or dizziness; (5) participants who have not had a cardiac arrest, angina pectoris or heart failure; and (6) participants with equal or greater than 6-score signs, symptoms or diseases using the previously validated PSIMP-RAA-10 tool [14].

#### Exclusion criteria

Participants with the following conditions will be excluded from the study: (1) being simultaneously involved in another research study; (2) heavy smokers (> 25 cigarettes/day), according to The Aspect Consortium of the European Commission classification scales [26]; (3) pregnant women; (4) receiving drug therapies with any type of antibiotic, antidepressant, sleeping pill, or anti-anxiety medication, or participants who will need to change their drug treatment protocol during the study; (5) previous diagnosis of coeliac disease (CeD), FA and/or FI, eating disorders, or infection due to the *Helicobacter pylori* bacteria during the last year; (6) participants with a job or lifestyle that potentially interferes with their regular sleep schedule (e.g., night shift and on-call); (7) participants with some type of major surgery that involves having been hospitalized during the last 5 years, including current muscle injuries and muscle cramps; (8) suffering from any type of cancer or other fatal illness within the last 5 years; (9) participants who are not willing to continue and finish the study for any reason; and (10) participants who, according to the researchers’ criteria, cannot continue the study (e.g., injuries, long trips, low commitment, outbreak of severe diseases, or similar).

#### Randomization

The ALASKA study will follow a sample randomization using the RedCap® randomization automated tool (RRAT) for the study allocation of subjects. RRAT assigns subjects by chance (rather than by choice) into specific groups (Group A and Group B) and monitors the overall allocation progress and assignment of randomized subjects. It is not possible to blind subjects or staff in this study from the tested treatment due to nutritional interventions which will be used as dietary treatments for the management of ARFS. Nevertheless, the outcome assessors will be blinded to the group assignment given the use of anonymized participant codes among all the steps of the study.

#### Allocation

Subjects will be randomly placed in Group A (control), or Group B (intervention) in a 1:2 allocation ratio using the RRAT tool. RRAT tool access will be granted to the outcome researchers of the Alaska study. The duration of the study for both control and intervention groups will be between 5 and 6 months. Individuals assigned to the control group will receive general nutritional advice with dietary recommendations for a healthy lifestyle (Annex I), while subjects assigned to the intervention group will receive a personalized food allergen-specific substitutive diet or FASSD (Annex II).

#### Intervention

The 6-month intervention of the ALASKA study consists of the implementation of a general nutritional advice with dietary recommendations for a healthy lifestyle (Annex I) during 5 to 6 months for subjects in the control group (Group A); and a 5/6-month FASSD (Annex II) for subjects in the intervention group (Group B) (Fig. 2). Nutrition and dietetics experts will elucidate the corresponding Annexes to the study participants during a one-to-one oral training session.

Annex I is based on the dietary guidelines for the Spanish population provided by the Spanish Society of Community Nutrition (SENC) and includes the following food groups: whole grain cereals, fruits, vegetables, oils and fats, meat, fish, legumes, nuts and seeds, eggs and dairy [27]. On the other hand, Annex II is based on substitutive foodstuffs according to national Spanish (Spanish Food Composition Database, BEDCA) [28] and international food databases (United States Department of Agriculture Food Composition Database, USDA-FCD and Nutrition Coordinating Center Food and Nutrient Database, NCCDB) [29, 30]. Foodstuffs with equal or higher predominant nutrients than the foodstuff to replace are chosen as equivalent foodstuffs.

Subjects in both groups (A and B), will be encouraged to follow the recommendations of Annex I. However, only Group B will be instructed to follow the personalized FASSD described in Annex II. Subjects in both groups will be followed up monthly, using an adherence monitoring tool (Annex IV. Dietary Adherence Questionnaire, DAQ, adapted to the 6-month intervention) during the ALASKA study (month 1 to month 5/6).

Regarding physical activity and sport habits, Annex I and Annex II request both groups (A and B) to maintain their usual physical activity and sport habits during the ALASKA study. Subjects will be instructed to make sure they accomplish the described conditions of the Inclusion and exclusion criteria form (Appendix 2) throughout this study.

#### Food allergen-specific substitutive diet

The FASSD (Annex II) is designed considering seven principal characteristics explained in Table 1. Substitutive foodstuffs of the FASSD are intended to be mainly a nutritional optimal replacement (concerning micronutrients of interest in the ARFS population); and only, when possible, a gastronomic substitutive belonging to the Mediterranean diet (MD). Instructions of the FASSD will be given as a printed catalog (Annex II). All the sections of this document will be explained to the corresponding subjects of study (Group B) verbally in a one-to-one oral training session.

**Table 1:** Design of a healthy food allergen-specific substitutive diet

Characteristic	Source	FASSD
(1) Essential food groups for a balanced diet	SENC Spanish nutritional pyramid [27]	whole grain cereals, fruits, vegetables, oils and fats, meat, fish, legume, nuts and seeds, eggs, and dairy
(2) Rich in lacking micronutrients of the ARFS population	literature [19, 20]	B2, B3, Mg, K, P, Ca, Zn, B12, B9, Fe, and fiber
(3) Foodstuffs with equal or greater micronutrients than the replaced foodstuff	BEDCA [28], USDA-FCD [29] and NCCDB [30] food composition databases	substitutive foodstuffs from the MD
(4) Positive immunological and enzymatic results	ALASKA study	Immunological: ≥ 3.5 kU_A_/l Enzymatic: H_2_ ≥ 20, CH_4_ ≥ 12 ppm changes
(5) Written session	ALASKA study	Printed report of the substitutive foodstuffs (Annex II) and the immunological and enzymatic results
(6) Oral session	ALASKA study	One-to-one personalized explanation of Annex II and the immunological and enzymatic results
(7) Monthly follow-up	DAQ (Annex IV)	Evaluation of the FASSD adherence

*ARFS*, adverse reactions to foodstuffs; *BEDCA*, Spanish Food Composition Database; *FASSD*, food allergen-specific substitutive diet; *MD*, Mediterranean Diet; *NCCDB*, Nutrition Coordinating Center Food and Nutrient Database; *USDA-FCD*, United States Department of Agriculture Food Composition Database. The FASSD was constructed according to the following references: SENC Spanish nutritional pyramid [27], micronutrients deficiency in ARFS population [19, 20], and national and international food composition databases [28–30]

#### Ethical considerations and clinical trial registration

This research will be performed in accordance with the Ethical Guidelines of the Declaration of Helsinki of 1964, revised in Fortaleza (2013) [31], and following the Spanish and European regulations on data protection [32]. The protocol has been approved by the Ethics Committee of the UPM (reference number 20200602, date: 17/07/2020) and registered on ClinicalTrials.gov (Clinical Trials ID NCT05802017, name: “Relation Between Adverse Reactions to Food, Physical Performance and Health in a Mediterranean Population”). A signed informed consent model will be obtained from all study participants before their participation commencement. As part of the information provided in the Informed consent model (oral and written information), participants will be informed about the researchers who will perform the study. The ALASKA study will be carried out by qualified professionals for medical, clinical, nutritional, and physical activity and sport sciences practice of the ImFINE-RG of the Department of Health and Human Performance of the Faculty of Physical Activity and Sport Sciences of the UPM (INEF-UPM). Besides, participants will be asked whether they authorize the researchers of this study to use their samples for subsequent genetic tests and/or future studies (see the “ Other variables” section).

In case of modification of the ALASKA study protocol (e.g., changes to eligibility criteria, outcomes, analyses, trial deviations, trial violations), the PI of the study, according to the favorable reports of the Ethics Committee of the UPM, will have to request a new protocol authorization, using the corresponding UPM official forms. After authorization and after receiving the new favorable reports, protocol modifications will be updated in the Clinical Trials registry. Protocol modifications will be communicated to the internal and external researchers involved in the ALASKA study (including the funding institution, in this case, the UPM) using the ‘File Repository’ section in RedCap®.

### Study variables

The following Table 2 displays a checklist example of a participant’s timeline with the assessments and instruments that will be considered during the ALASKA study. The following table meets The Standard Protocol Items: Recommendations for Interventional Trials (SPIRIT) [24].

**Table 2:**
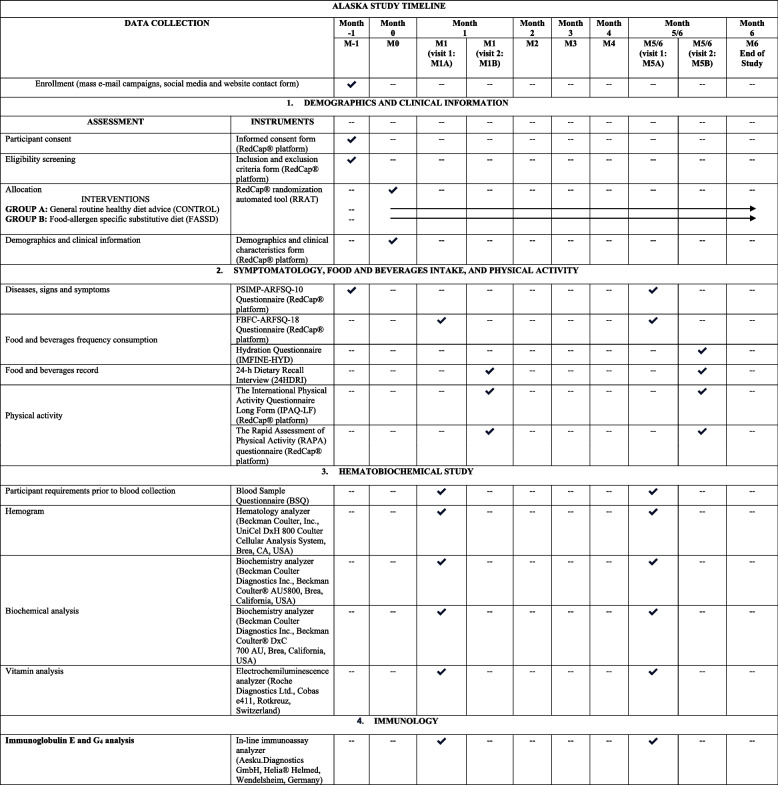

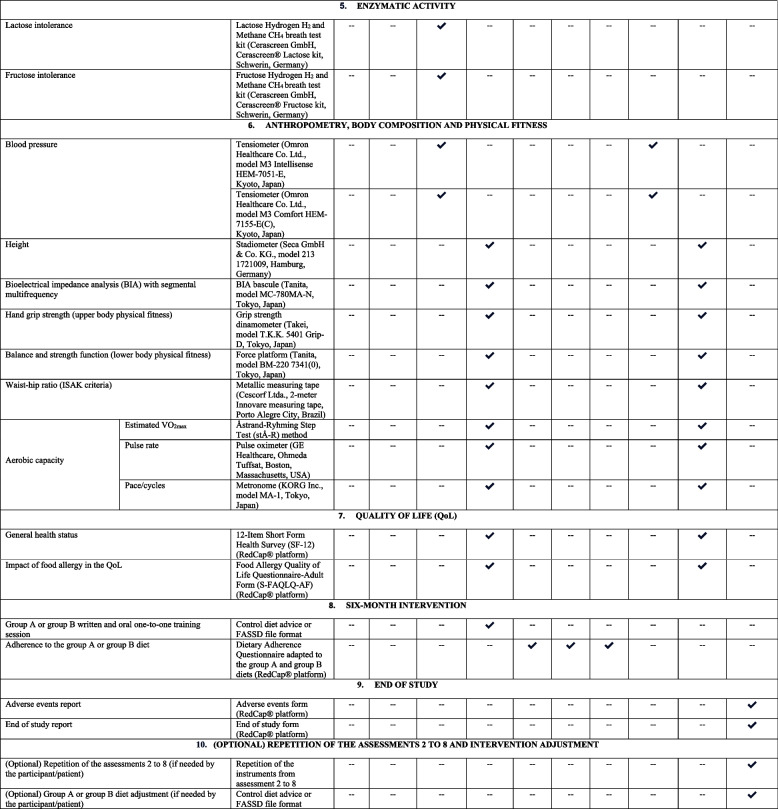
Checklist example of a participant’s timeline with assessments and instruments during the ALASKA study

*BIA*, bioelectrical impedance analysis; *FASSD*, food allergen-specific substitutive diet; *FBFC-ARFSQ-18*, Food and Beverages Frequency Consumption Questionnaire to Identify Adverse Reactions to Foodstuffs; *IMFINE-HYD*, ImFINE Research Group Hydration Questionnaire; *IPAQ-LF*, The International Physical Activity Questionnaire Long Form; *ISAK*, International Society for the Advancement of Kinanthropometry; *H*_*2*_, hydrogen; *CH*_*4*_, methane; *M − 1*, month 1; *M0*, month 0; *M1*, month 1; *M2*, month 2; *M3*, month 3; *M4*, month 4; *M5/6*, month 5 or month 6; *M5*, month 5; *M6*, month 6; *M1A*, visit 1 at month 1; *M1B*, visit 2 at month 2; *M5A*, visit 1 at month 5 or month 6; *M5B*, visit 2 at month 5 or month 6; *RAPA*, The Rapid Assessment of Physical Activity Questionnaire; *RRAT*, RedCap® randomization automated tool; *RedCap®*, Research Data Capture Platform; *PSIMP-ARFSQ-10*, Pathologies and Symptomatology Questionnaire associated with Adverse Reactions to Foodstuffs; *QoL*, quality of life; *S-FAQLQ-AF*, Food Allergy Quality of Life Questionnaire-Adult Form; *SF-12*, 12-Item Short Form Health Survey; *VO*_*2max*_, maximal oxygen consumption

#### Main variables

**Fig. 3:**
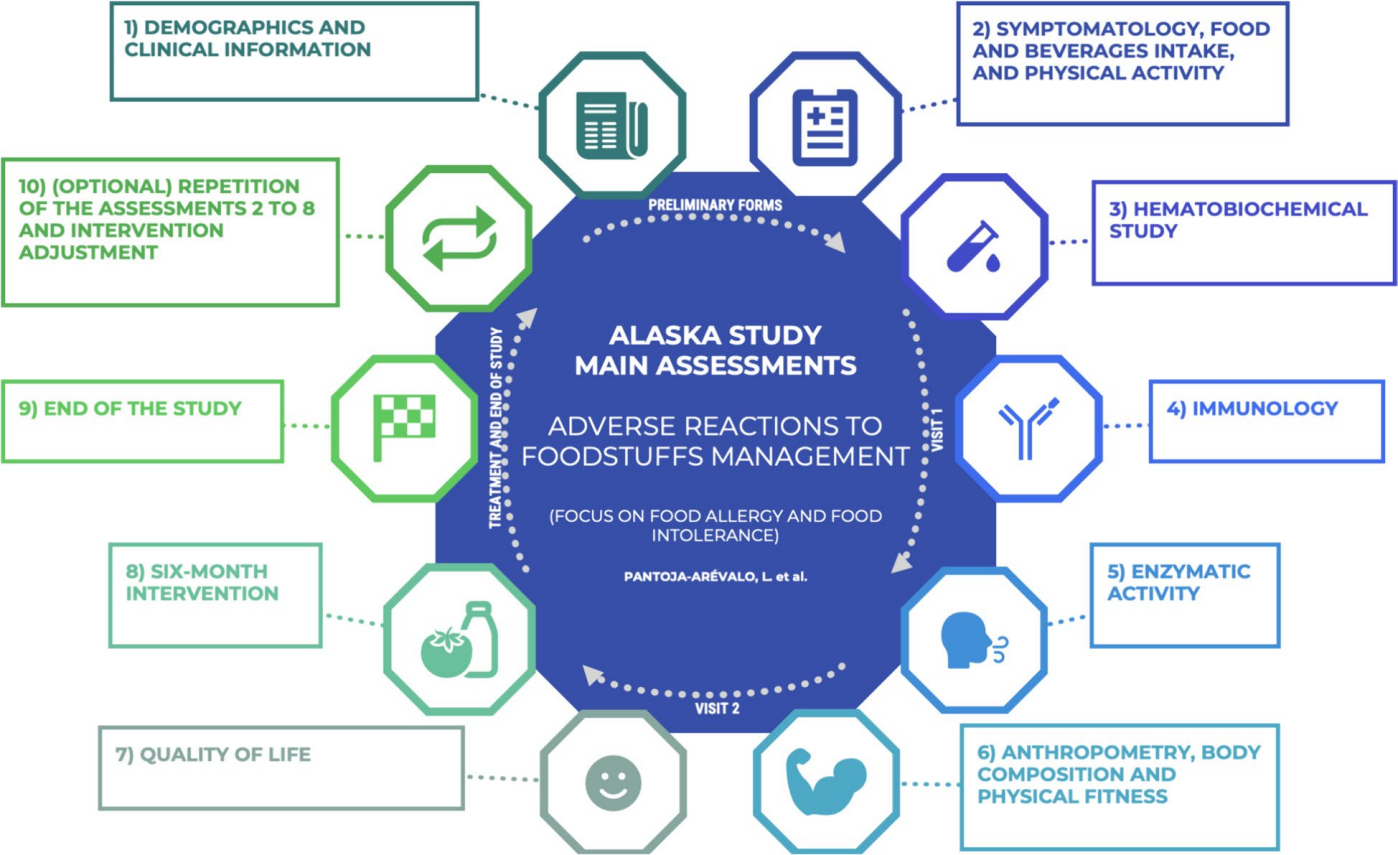
ALASKA study main variables organized as 10 main assessments for the adverse reaction to foodstuffs management

The ALASKA study, as a proposed global strategy to manage ARFS in the Spanish adult population, is organized in the following main variables described as 10 main assessments (Fig. 3).

## Demographics and clinical information

Demographics and clinical characteristics presented in Table 3 will be collected from all the selected subjects of the study. Information about the latest SARS-CoV-2 vaccine (last 3 months), brand, number of received doses, and the result of the most recently performed coronavirus disease 2019 (COVID-19) test will be gathered. Additional clinical information will be possible to gather using an optional open text box of other relevant clinical information as part of the demographics and clinical characteristics form.

**Table 3:** Demographics and clinical information

Instrument	Variable	Description
Demographics and clinical characteristics form	Age	Years of age
	Sex	Men, women
	Country of birth	Country of origin
	Home city	Place of residence
	Level of education	Without education, school, high school, professional training medium/superior level, university, master and doctorate levels
	Employment	Self-employed or employed, student paid/unpaid, unemployed, retired or pre-retired, permanently disabled, houseworker
	Occupation	Profession
	Number of people living together	Number of people living together, in the same residence, with the subject of study
	Blood type	A + , A − , B + , B − , O + , O − , AB + , AB −
	SARS-CoV-2 test	Type, brand, and result (p/n)
	SARS-CoV-2 vaccine	y/n, doses and brand
	(Optional) additional relevant clinical information	Open text box

*p/n*, positive or negative; *SARS-CoV-2*, severe acute respiratory syndrome coronavirus 2; *y/n*, yes or no

## Symptomatology, food and beverages intake, and physical activity

The validated Pathologies and Symptomatology Questionnaire associated with Adverse Reactions to Foodstuffs (PSIMP-RAA-10); and Food and Beverages Frequency Consumption Questionnaire to Identify Adverse Reactions to Foodstuffs (FBFC-ARFSQ-18) tools will be used to record the clinical picture (diseases, signs and symptoms) and the food frequency consumption of the subjects of study [14]. Additionally, hydration status will be assessed using the Hydration Questionnaire of the ImFINE Research Group (IMFINE-HYD) to record water and other beverage intake [33]. Besides, food and beverages record will be measured through a 24-h dietary recall interview (24HDRI) (Annex III). Medication and food supplement intake will be also assessed in this interview. The daily energy and nutrient intake of each participant will be calculated by using DIAL software [34], which works with food composition databases such as the Spanish Food Composition Database (BEDCA) and the United States Department of Agriculture Food Composition Database (USDA-FCD), among others. Additionally, to encourage subjects to follow the assigned 6-month intervention and promote the dietary treatment adherence, all subjects will be contacted monthly during the study using a Dietary Adherence Questionnaire (Annex IV, authors’ own creation, based on questions of previously published validated questionnaires [35] and adapted to the 6-month intervention). Subjects will be asked to maintain their normal physical activity habits during the time of the study, from M1 to M5/6.

The International Physical Activity Questionnaire Long Form (IPAQ-LF) [36] and the Rapid Assessment of Physical Activity (RAPA) [37] will be administered to determine the minutes per week of self-reported physical activity and the physical activity scores of the study sample, respectively. Using the IPAQ-LF, evaluations are made according to the types of physical activities that people do as a part of their daily lives (walking, moderate to severe information about time spent in activities, and time spent sitting). The energy required will be calculated with the MET (metabolic equivalent)-minute scores.

## Hematobiochemical study

Venous blood samples will be collected in fasting conditions (at least 8 h), and after completing the Blood Sample Questionnaire (BSQ) (Annex V. BSQ adapted for the ALASKA study). Blood samples will be collected into one 3-ml tube containing dipotassium (K2) ethylene diamine tetraacetic acid (EDTA) (K2-EDTA Vacutainer® BD tube, REF367838) (BD Becton, Dickinson and Company NYSE: BDX, Sunnyvale, CA, USA); two 5-ml tubes containing separating gel (Vacutainer® BD serum-separating tube SST™ II Advance, REF368968) (BDX, Sunnyvale, CA, USA); and one 4-ml tube containing lithium heparin (LH Vacutainer® BD tube, REF368884) (BDX, Sunnyvale, CA, USA).

Complete blood count (CBC) will be performed using a hematology analyzer, UniCel DxH 800 Coulter Cellular Analysis System (Beckman Coulter, Inc., Brea, CA, USA), with included impedance, radio frequency (RF), flow cytometric light scatter, spectrophotometry, and supravital staining techniques to be applied in the subjects’ whole blood K2-EDTA tubes. Quantitative determination of the following hematological parameters will be tested: erythrocyte series (red blood cell count), leukocyte series (white blood cell count), and platelets.

After separating the serum by centrifugation, the samples will be measured for biochemical analysis of glucose, cholesterol (total, HDL, and LDL), triglycerides, serum albumin, urate, creatinine, urea, sodium, potassium, chloride, bilirubin, aspartate aminotransferase, alanine aminotransferase, gamma-glutamyl transferase, alkaline phosphatase, creatine kinase, iron, calcium, magnesium, lactate dehydrogenase, amylase, and lipase. Biochemical analysis will be measured using two clinical chemistry analyzers through the spectrophotometric method, Beckman Coulter® AU5800 (Beckman Coulter Diagnostics Inc., Brea, California, USA) and Beckman Coulter® DxC 700 AU (Beckman Coulter Diagnostics Inc., Brea, California, USA).

Lastly, the vitamin profile will be determined through electrochemiluminescence of the Cobas e411 (Roche Diagnostics Ltd., Rotkreuz, Switzerland). Remaining samples from subjects who offer their consent (serum, plasma, and whole blood) will be stored in 1 ml aliquots at − 80 °C (ultrafreezing conditions). Colored 2-ml microtubes will be used for the ultrafreezing storage of 1 ml aliquots of serum (yellow, REF05-408–140), plasma (green, REF05-408–142) and whole blood (red, REF05-408–139) (Thermo Fisher Scientific Inc., Waltham, Massachusetts, USA).

## Immunology

Quantitative detection of food-specific IgE and IgG_4_ antibody (Ab) reactions, in kilounits of allergen-specific IgE or IgG_4_ per liter (kU_A_/l), against coated food antigens will be determined in human serum samples (Table 4). Ready-to-use AESKUBLOTS® kits will be performed using the immunoblot multiplex technology of the Helia® Helmed Line Immunoassay analyzer (Aesku.Diagnostics GmbH, Wendelsheim, Germany). Positive Ab reactions will be considered as ≥ 3.5 kU_A_/l. In vitro tests, specifically molecular-based allergy diagnostics with multiplexed technology, allow researchers to define the immunoglobulin profile of interest of the study sample. This approach is in line with the Precision Medicine Statements [38].

**Table 4:** Food allergen panels in type I and type II food allergy for the detection of IgE and IgG_4_ Ab reactions in human serum samples

ARFS	Marker	AESKUBLOTS® kit (reference/name)	Food allergen panel
**T1FA**	IgE	421,001/ E-5	Egg white (f1), egg yolk (f75), milk (f199), cod (f3), wheat (f4), soybean (f14), peanut (f13), cashew nut (f204), crab (f23), shrimp (f24), beef (f27), lamb (f88), tomato (f25), pineapple (f52), mango (f91), and total IgE (IgE)
		421,011/ E-11	Tomato (f25), carrot (f31), celery (f85), mustard (f89), garlic (f47), onion (f48), banana (f29), orange (f33), strawberry (f44), apple (f49), peach (f53), kiwi fruit (f84), pineapple (f52), mango (f91), egg white (f1), milk (f199), cod (f3), salmon (f41), tuna (f40), shrimp (f24), beef (f27), lamb (f88), chicken (f83), wheat (f4), barley (f6), oat (f7), maize (f8), rice (f9), sesame (f10), soybean (f14), peanut (f13), bean, green (f950), potato (f35), ginger (s15, walnut (f16), hazelnut (f17), almond (f20), cashew nut (f204), CCD (CCD), and total IgE (IgE)
**T2FA**	IgG_4_	431,401/ G4-1	Salmon (f41), cod (f3), herring (f21), shrimp (f24), blue mussel (f37), pork (f26), beef (f27), chicken (f83), wheat (f4), rye (f5), oat (f7), maize (f8), rice (f9), buckwheat (f11), amaranth (f811), carrot (f31), celery (f85), cabbage (f39), garlic (f47), onion (f48), pea, green (f12), bean, green (f950), soybean (f14), peanut (f13), tomato (f25), cucumber (f120), potatoe (f35), mustard (f89), egg white (f1), egg yolk (f75), milk (f199), goat´s milk (f300), sheep´s milk (f325), orange (f33), banana (f29), apple (f49), kiwi fruit (f84), hazelnut (f17), walnut (f16), and almond (f20)
		431,402/ G4-2	Salmon (f41), cod (f3), plaice (f152), squid (f176), blue mussel (f37), octopus (f819), pork (f26), beef (f27), lamb (f88), wheat (f4), oat (f7), sesame (f10), spelt (f158), amaranth (f811), carrot (f31), cucumber (f120), broccoli (f182), garlic (f47), maize (f8), cabbage (f39), celery (f85), sweet lupine (f827), egg white (f1), egg yolk (f75), cheese, gouda (f200), casein (f78), apple (f49), orange (f33), grape (f50), peach (f53), mango (f91), almond (f20), hazelnut (f17), peanut (f13), walnut (f16), pistachio nut (f818), cashew nut (f204), potato (f35), baker’s yeast (f45),and soybean (f14)
		431,403/ G4-3	Tuna (f40), trout (f930), pollock (f802), herring (f21), oyster (f177), shrimp (f24), duck (f58), chicken (f83), turkey (f143), rye (f5), buckwheat (f11), barley (f6), durum wheat (f159), millet (f164), quinoa (f832), gluten (f79), tomato (f25), onion (f48), zucchini (f197), olive, green (f812), lentil (f65), pea, green (f12), bean, green (f950), milk (f199), sheep´s milk (f325), goat´s milk (f300), banana (f29), kiwi fruit (f84), lemon (f32), strawberry (f44), pineapple (f52), sunflower seed (f156), pumpkin seed (f157), mustard (f89), sweet basil (s11), ginger (s15), coffee (f955), cacao (f97), button mushroom (f141), and rice (f9)

*ARFS*, adverse reactions to foodstuffs; *CCD*, cross-reactive carbohydrate determinants; *IgE*, immunoglobulin E; *IgG*_*4*_, immunoglobulin G_4_; *T1FA*, type I food allergy; *T2FA*, type II food allergy

## Enzymatic activity

Hydrogen (H_2_) and methane (CH_4_) concentrations will be measured in the participant’s exhaled air for the assessment of lactose intolerance and fructose malabsorption. Ready-to-use Cerascreen® lactose and fructose substrate kits will be used (Cerascreen GmbH, Schwerin, Germany). Cerascreen® kits consist of a single-use blower device, 5 breath-sample collector glass tubes, and 20 g of the substrate. Before taking the breath test, subjects will need to meet specific test requirements (Annex VI. Breath test instructions), such as fasting conditions (at least 8 h) and a restrictive diet consisting of white rice, meat (fish, beef, chicken, or egg), extra virgin olive oil and water (water, tea or black coffee). Subjects will be able to perform the breath tests at home following the steps of Annex VI.

In order to collect the breath samples, subjects will drink a solution of 20 g of the substrate (lactose or fructose). Then, breath samples will be collected every 30 min up to 120 min (5 glass tubes). The first tube will be saved for a blank breath sample collection before drinking the solution (control tube). The concentration of exhaled gases (H_2_/CH_4_) will be quantified using the BreathTraker® gas breath analyzer (QuinTron Instrument Company Inc., Milwaukee, WI, USA). Results will be read according to the following criteria: changes of H_2_ ≥ 20 ppm and CH_4_ ≥ 12 ppm as positive results.

## Anthropometry, body composition, and physical fitness

### Anthropometry

Body weight (BW) will be measured in fasting conditions with an electronic calibrated MC-780MA-N Tanita bascule device (Tanita, Tokyo, Japan). Height will be assessed using a 2-m stadiometer Seca 213 1,721,009 (Seca GmbH & Co. KG., Hamburg, Germany) in a standing position and vertical plane. Subjects will be without shoes and with heels against the stadiometer base wall. Feet will be together, and knees will be straight and without bending. Subjects will be looking straight ahead with head in the Frankfort horizontal plane position (the lower margin of the eye bony socket aligned with the upper margin of the external auditory meatus). Subjects will be asked to breathe slowly during height measurements. Height will be recorded twice with an accuracy of 0.1 cm. Body mass index (BMI) will be calculated using body weight in kilograms (kg) divided by height in meters squared (m^2^), kg/m^2^ [39].

Waist-hip ratio will be calculated according to The International Society for the Advancement of Kinanthropometry (ISAK) using the 2-m Innovare metallic measuring tape (Cescorf Ltda., Porto Alegre City, Brazil).

### Body composition

Multi-frequency segmental body composition will be performed using bioelectrical impedance analysis (BIA) using the MC-780MA-N Tanita device (Tanita, Tokyo, Japan). The analysis will include body fat percentage (BFP), body fat mass (BFM), fat-free mass (FFM), basal metabolic rate (BMR), total muscle mass (MM), mineral bone mass (MBM), skeletal muscle mass (SMM) and total body water (TBW). Before the test, subjects will have to accomplish particular requirements: 8-h fasting condition, wear comfortable clothes avoiding adjusted fabrics, avoid body unguents and having wet skin or hair, avoid alcoholic beverages during the previous 24 h, avoid difficult to digest foodstuffs (dairy, bakery, vinegar, or soy sauce) during the previous 12 h, urinate 30 min before the test; and avoid physical activity that may provoke cramps during the previous 12 h. Subjects will place their feet on top of the electrodes so that the heel is central to the smaller posterior electrode and the forefoot is central to the larger anterior electrode. Hands will touch the electrodes so that the electrode separator is positioned between the middle and ring finger. Duplicate measurements will be saved and stored in a secured digital (SD) memory card.

### Physical fitness: cardiorespiratory fitness test, equilibrium test and handgrip strength

#### Cardiorespiratory fitness test

Cardiorespiratory fitness (CRF) will be determined by estimating the maximal oxygen consumption (VO_2max_) using the Åstrand-Ryhming Step Test (stÅ-R) [40, 41]. VO_2max_ will be predicted according to Å-R’s nomograms by the determination of submaximal heart rate (HR) values using a professional oximeter device (General Electric, GE, Ohmeda Tuffsat) (GE Healthcare, Boston, Massachusetts, USA). HR will be recorded every minute during a total test time of 5 min. Final HR will be recorded as the maximal value during the last 30 s of the submaximal fifth minute. For the final reading of the estimated VO_2max_, Å-R’s nomograms will consider the participant’s final HR, sex, age, and weight.

The stÅ-R will be performed using a 40-cm and 33-cm height step for men and women, respectively. A MA-1 metronome (KORG Inc., Tokyo, Japan) will be set up at 22.5 cycles per minute to help subjects maintain a standard stepping pace. All measurements will start with a two-step warm-up to check the ability of the participant using the assigned height and step pace. The American Heart Association (AHA) classification [42] will be used to determine the categories of the estimated VO_2max_ and to subsequently organize the sample in low, normal, and excellent aerobic capacity.

#### Equilibrium test

Power, speed, and balance (chair sit-to-stand test) will be measured using the Tanita BM-220 force platform and the Zaritz software (Tanita, Tokyo, Japan). Sarcopenia assessment, lower extremity function, and physical fitness (PF) will be evaluated. Subjects will sit on a 40-cm-height standard chair with their barefoot, legs shoulder-width apart, the trunk stretched vertically in a straight line, and their ankles held at 90°. Following the instructions of the Zaritz software, subjects will have to sit and stand 3 consecutive times from the chair. Values of a single try of 3 times will be recorded.

#### Handgrip strength

Handgrip strength (HGS) will be measured, using a digital 5401 Grip-D dynamometer (Takei Scientific Instruments Co. Ltd, Tokyo, Japan). Muscle strength, upper extremity function and PF will be evaluated. Before the performance of the test, the dynamometer regulation will be set at 3.5 and 4 cm according to the participant’s size between their phalanges. HGS will be performed in both hands, right and left, while sitting (40-cm-height standard chair) and standing (supine position). Subjects will rest for 30 s between each measurement and the highest values will be recorded after duplicate testing.

### Quality of life

The self-perceived health-related QoL of the subjects of study will be assessed using the validated tools: Short-Form Health Survey 12 (SF-12) and the Spanish validated version of the Food Allergy Quality of Life Questionnaire—Adult Form (S-FAQLQ-AF).

### Six-month intervention

Once the results of the blood samples are acquired, the expert-participant meetings will be arranged. During a one-to-one oral training session of approximately 15 min, Annexes I and II will be discussed and explained to the corresponding group of subjects. Annex I will be explained to the control group (Group A) and Annexes I and II, to the intervention group (Group B). Positive (> 3.5 kU_A_/l) foodstuffs from serum (sIgE) and sIgG_4_ AbR blood tests will be replaced with alternative food items listed in Annex II (FASSD). Adherence to the FAASD will be checked on a monthly basis (Annex IV. Dietary Adherence Questionnaire, DAQ) and any adverse event, missing data, or dropouts will be documented during the 6-month intervention (Annex VII. Adverse Event Form and Annex VIII End of Study Form).

### End of the study and adverse events

Any potential negative outcomes linked to the dietary changes (minimal to zero risk probability) of the ALASKA study intervention, if any, will be documented using Annexes VII and VIII. The UPM will assist with an available medical physician, part of the ALASKA study researchers (M.P.-R.), to cover for any emergency associated with any step of this study protocol and will be present during all the clinical and physical assessments.

### (Optional) Repetition of the assessments 2 to 8 and intervention adjustment.

The ALASKA study focuses mainly on a 6-month intervention/follow-up. However, the influence of ARFS and the effect of a FASSD in health and QoL may be long term. At the end of the trial, if the study budget allows, patients will have the opportunity to undergo a re-evaluation for all main variables from 1 to 8. Nevertheless, they will always be able to visit the INEF-UPM and get evaluated for variables with non-consumable measurement instruments, such as variables from 1 to 2 and 6 to 8 (Fig. 3).

Therefore, as an optional step, assessments will be repeated at M5/6 according to the established ALASKA study timeline described in Table 2. Food-specific sIgE and sIgG_4_ Ab reactions, as well as its corresponding substitutive foodstuffs, may change according to the new results of the immunological food profile and/or enzymatic tests. Following another consecutive 6-month intervention, using the recommendations of Annex I and Annex II instructions and based on the new results, will be suggested as an optional step. If no optional step will be taken by the subjects of study, they will be advised by a qualified professional of the nutritional practice on the adequate steps to be followed to retake a common healthy balanced diet (Dietary Guidelines for the Spanish population provided by the Spanish Society of Community Nutrition (SENC) [27].

### Other variables

When analyzing other types of ARFS, apart from FA or FI, such as CeD, there could be other recommended relevant variables of interest. Some of these variables include the characterization of the microbiota, genetic testing (HLA DQ-2, DQ-8), and serum biomarkers such as type 2 transglutaminase (TG2), deamidated gliadin peptide (DGP), gliadins, microbial transglutaminase (mTG), and mTG neo epitopes [43, 44].

### Statistical analysis

The sample size required for the current study was calculated using G*Power software v.3.1. (Heinrich-Heine-Universität Düsseldorf, Düsseldorf, Germany) [25]. All subjects of study with complete data will be analyzed. First, the normal distribution of the data will be checked using the Kolmogorov–Smirnov test. In case of the non-normal distribution of a variable, a logarithmic transformation will be conducted. Independent sample’s *t*-test (or its non-parametric Mann–Whitney *U* test) will be used to compare the baseline values of continuous variables between the two groups (A and B). Moreover, the chi-square test will be conducted to compare categorical variables. To examine the effect of the FASSD on the outcome variables, we will apply repeated measures analysis of variance (RM-ANOVA), in which the effect of time, intervention, and time*intervention interaction will be explored. Changes in outcome variables will also be examined between the two groups using an analysis of covariance (ANCOVA), in which the baseline values will be considered as covariates. A strong exclusion criteria list will be used in the ALASKA study to reduce confounding variables from interfering in the effect analysis. However, to analyze the multifactorial approach of multiple influencing variables over the ARFS together with the effect of the FASSD, the selection of confounding variables (dietary habits, QoL, sampling time and trial season, medication or food supplement intake), other than baseline characteristics, will be also considered. Corresponding Odds ratio (OR), or when justified Mantel–Haenszel (M-H) estimators will be used together with logistic or liner regressions, or ANOVA and ANCOVA tests, as it may apply for the corresponding variable.

Valid data values will be considered of subjects of study accomplishing at least main variables from 1 to 4 and from 6 to 7 (see the “ Main variables” section), the rest of the data values will be carefully either cleaned or imputed as missing values. Data will be analyzed using IBM-SPSS® Statistics software, version 26.0, and statistical significance will be set at 0.05.

## Discussion

ARFS is a current prevalent complication and chronic disease, which is continuously raising specially in patients with specific related-diseases and symptomatology. ARFS management approaching a multivariable model, including most influencing factors of FA and FI, can be used to evaluate ARFS from a wider perspective.

Demographic variables are considered a key predictor of literacy, QoL, and health in the clinical research and practice of chronic diseases. Age and sex may lead to different clinical outcomes, such as signs and symptoms, depending on specific pathologies and chronic diseases [14]. The country of birth, home city, or district may signify an essential determinant of health given that mortality, healthcare, culture, politics, socio-economics, and environment will be directly related to the participant’s place of birth and living [45]. Education and occupation, as skill predictors, may signify key factors in chronic diseases involving the NS (e.g., memory, aptitude, ability, vitality) [46]. Employment has been also considered an influencing factor of the QoL in several chronic diseases involving the NS [47]. The number of people living together, in the same residence, can be optimal to measure the dynamics of relationships, as relevant sources of support, at different age stages of adulthood [48]. The identification of clinical variables is essential when investigating chronic diseases because, combined with demographic variables, they represent a consistent measurement of health and QoL [49].

ARFS may have a significant influence in the QoL and health, especially in individuals with FA or FI [50]. Lactose intolerance and fructose malabsorption (FIs) have been associated with poorer perceived QoL and health [51, 52]. Furthermore, studies have agreed that there is a lack of information and relatively little is known regarding the impact on QoL and health of individuals with FI. On the other hand, despite recent consensus on the FA detection procedures (IgE Ab reactions) and treatment (strict allergen avoidance); the necessity to expand the potential biomarkers for ARFS research and the need of novel proposals of nutritional treatments that could avoid micronutrient deficiencies is still latent [4]. IgG subclass 4 Ab reactions might be of interest when studying food and environmental allergens and its significance to specific types of diseases and symptoms (e.g., irritable bowel syndrome [53], allergic rhinitis [54], and chronic pain [55]).

Non-strict and partial avoidance diets (encouraging the substitutive dietary treatment proposal) have suggested possible improved hematobiochemical and immunological profiles, anthropometry, body composition, and muscle strength [56]. Review studies have observed that a complete exclusion of dairy products may favor the development of bone diseases such as osteopenia, osteoporosis, and muscle sarcopenia [57]. In clinical trials, the strict avoidance of positive food-specific IgG_4_/IgE Ab reactions against food allergen mixtures has improved irritable bowel disease (IBD) symptomatology [58], atopic dermatitis [59], and mental illnesses [60]. Weight and blood dyscrasias seem to be other key features in ARFS, weight loss, and abdominal pain seem to be tightly related in FI-positive profiles [61].

To our knowledge, there is no further clinical trial examining the effect of the FASSD, or similar, on symptomatology, nutritional status, hematobiochemical and immunological makers, enzymatic activity, anthropometry, body composition, and QoL of patients with ARFS. Considering that muscle mass and strength, nutritional status, and QoL of patients with ARFS are being influenced due to strict food avoidance diets, it is expected that this type of dietary treatment, the FASSD, might improve the nutritional status of patients with ARFS. The logic behind such hypothesis was the particular main characteristic of the FASSD which includes the micronutrients that are generally deficient in individuals with FA or FI, and through which it may affect QoL and health.

### Strengths and limitations

This study proposes a novel methodological tool for the evaluation and management of ARFS considering a variety of influencing factors such as the symptomatology, food and beverages intake, hematobiochemical and immunological studies, enzymatic activity, physical fitness, anthropometry and body composition. This study proposes the first RCT on the effect of a substitutive diet on symptomatology, nutritional status, hematobiochemical and immunological makers, enzymatic activity, anthropometry, body composition, and QoL among patients with ARFS. The findings of this study will provide evidence on the protocol.

## Conclusions

The ALASKA study protocol has been developed as a global strategy to manage and evaluate ARFS (particularly FA and FI) in Spanish adults older than 18 years of age. Approaching ARFS with multiple assessments, as influencing factors, such as symptomatology, food and beverages intake, hematobiochemical and immunological studies, enzymatic activity, physical fitness, anthropometry, and body composition, will lead to a novel strategy for ARFS management and the evaluation of its impact on QoL and health. The FASSD has been designed as a personalized tool to avoid crucial micronutrient deficiencies that a current strict food allergen avoidance or elimination diet may provoke. The FASSD considers essential food groups for a balanced diet and includes special attention to B2, B3, Mg, K, P, Ca, Zn, B12, B9, Fe, and fiber. The ALASKA study protocol can be used, in the research and clinical practice, for the investigation and evaluation of general ARFS and to seek its relationship with related diseases, symptomatology, food and beverages intake, physical fitness, anthropometry, body composition and novel potential food-specific biomarkers.

## Trial status

Recruitment of participants started in April 2022 and finished May 2023. Earlier study protocol submission was not possible because of protocol adaptations due to COVID-19 pandemic. The ALASKA study is still ongoing now and fieldwork is foreseen to be finished by July 2024.

### Supplementary Information

Additional file 1: Supplementary Information (Annex I to VIII). Annex I. General nutritional advice with dietary recommendations for a healthy lifestyle. Annex II. Instructions of the food-allergen specific substitutive diet (FASSD). Annex III. 24-hour dietary recall interview (24HDRI). Annex IV. Dietary Adherence Questionnaire (DAQ, adapted to the ALASKA study). Annex V. Blood Sample Questionnaire (BSQ) adapted to the ALASKA study. Annex VI. Breath test instructions. Annex VII. Adverse Event Form. Annex VIII. End of Study Form.

Additional file 2. SPIRIT checklist.

Additional file 3: Appendix 1. Informed consent model for the ALASKA study.

Additional file 4: Appendix 2. Inclusion and exclusion criteria form.

## Abbreviations

Ab: Antibody
AHA: American Heart Association
ALASKA study: Allergies and Food Intolerances in Adults and Athletes. UPM study project: Relation between adverse reactions to food, physical performance and health in a Mediterranean population. The ALASKA study
ARFS: Adverse reactions to foodstuffs
BD: Becton Dickinson
BEDCA: Spanish Food Composition Database
BIA: Bioelectrical impedance analysis
BMI: Body mass index
BSQ: Blood Sample Questionnaire
CeD: Coeliac disease
CESVIMA: Supercomputing and Visualization Center of Madrid Community
CBC: Complete blood count
COVID-19: Coronavirus disease 2019
CRC: Clinical research coordinator
CRF: Cardiorespiratory fitness
DAQ: Dietary Adherence Questionnaire
DGP: Deamidated gliadin peptide
EAACI: European Academy of Allergy & Clinical Immunology
EDTA: Ethylene diamine tetraacetic acid
e.g.: Exempli gratia (latin), for example
FA: Food allergy
FASSD: Food allergen-specific substitutive diet
FBFC-ARFSQ-18: Food and Beverages Frequency Consumption Questionnaire to Identify Adverse Reactions to Foodstuffs
FI: Food intolerance
HGS: Handgrip strength
HLA: Human leukocyte antigen
HR: Heart rate
i.e.: Id est (latin), that is, in other words
IgE: Immunoglobulin E
IgG4: Immunoglobulin G4
IHR: Immune-mediated immediate hypersensitivity reactions
INEF-UPM: Faculty of Physical Activity and Sport Sciences of the UPM
ImFINE-RG: ImFINE Research Group
IMFINE-HYD: Hydration Questionnaire of the ImFINE Research Group
IPAQ-LF: The International Physical Activity Questionnaire Long Form
ISAK: International Society for the Advancement of Kinanthropometry
kUA/l: Kilounits of allergen-specific IgE or IgG4 per liter
K2: Dipotassium
LH: Lithium heparin
mTG: Microbial transglutaminase
M1: Month 1 of the ALASKA study
M5/6: Month 5 or 6 of the ALASKA study
NCCDB: Nutrition Coordinating Center Food and Nutrient Database
NS: Nervous system
OFC: Oral food challenge
PAL: Physical activity level
PF: Physical fitness
PPM: Parts per million
PSIMP-RAA-10: Pathologies and Symptomatology Questionnaire associated with Adverse Reactions to Foodstuffs
QoL: Quality of life
RAPA: The Rapid Assessment of Physical Activity Questionnaire
RCT: Randomized controlled trial
RedCap®: Research Electronic Data Capture Platform
RF: Radio frequency
RRAT: RedCap® randomization automated tool
SENC: Spanish Society of Community Nutrition
SF-12: Short-Form Health Survey 12
S-FAQLQ-AF: Spanish validated version of the Food Allergy Quality of Life Questionnaire-Adult Form
SPIRIT: Standard Protocol Items: Recommendations for Interventional Trials
stÅ-R: Åstrand-Ryhming Step Test
T1FA: Type I hypersensitivity/ food allergy
T2FA: Type II hypersensitivity/ food allergy
TG2: Type 2 transglutaminase
UPM: Universidad Politécnica de Madrid
USDA-FCD: United States Department of Agriculture (USDA) Food Composition Database
VO_2max_: Maximal oxygen consumption
WHO: World Health Organization
24HDRI: 24-h dietary recall interview
