# Supplementary material for: The multifactorial approach and the food allergen-specific substitutive diet as a tool to manage and ameliorate adverse reactions to foodstuffs in adulthood: study protocol for a randomized controlled trial—the ALASKA study
Source: Trials. 2024 Jul 20;25:494. doi: 10.1186/s13063-024-08307-2 (PMC11264907; doi:10.1186/s13063-024-08307-2)
Supplement: Supplementary file 1 — Additional file 1: Supplementary Information (Annex I to VIII). Annex I. General nutritional advice with dietary recommendations for a healthy lifestyle. Annex II. Instructions of the food-allergen specific substitutive diet (FASSD). Annex III. 24-hour dietary recall interview (24HDRI). Annex IV. Dietary Adherence Questionnaire (DAQ, adapted to the ALASKA study). Annex V. Blood Sample Questionnaire (BSQ) adapted to the ALASKA study. Annex VI. Breath test instructions. Annex VII. Adverse Event Form. Annex VIII. End of Study Form. [file 13063_2024_8307_MOESM1_ESM.pdf]

ANNEX I. GENERAL NUTRITIONAL ADVICE WITH DIETARY RECOMMENDATIONS FOR A HEALTHY LIFESTYLE

INSTRUCTIONS:

It is recommended to consume, **at least the described number of servings per week and per day** (section B "SECOND LEVEL OF THE PYRAMID"), of the food and beverages from each food group. In its place, if you are unable to consume any of the described food or beverages, it is recommended to consume its substitute food or beverage (Annex II). If you have not received Annex II, please follow the recommendations in this Annex only (Annex I).

If you have any questions regarding these recommendations, please contact the research staff at l.pantoja@upm.es, marcela.gonzalez.gross@upm.es, or gi.imfine@upm.es.

According to the dietary guidelines for the Spanish population of the Spanish Society of Community Nutrition (SENC) updated in 2019 [1], the corresponding recommendations are explained below (Figure 1).

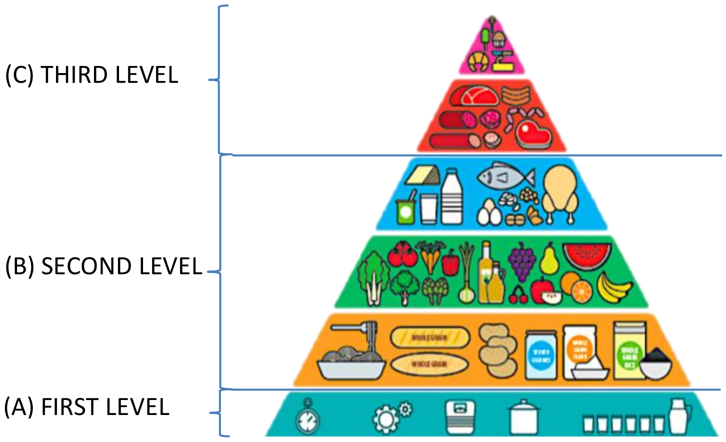

Figure 1 Healthy Eating Pyramid. Spanish Society of Community Nutrition (SENC), 2019 [1]

A. FIRST LEVEL OR BASE OF THE PYRAMID:

- Keep adequate levels of daily **physical activity** to maintain good health and improve quality of life (60 minutes a day of moderate-intensity physical activity, or the equivalent of 10000 steps a day, with some occasional steps or periods of higher intensity). **Note:** this present study is not focused in physical activity interventions; therefore, **maintain your usual physical activity and sport habits** during the time of this present dietary treatment (5 to 6 months).
- Dedicate time to food shopping and cooking, as well as to share this knowledge to those closest individuals (promotion of **emotional balance**).
- Adjust the food intake to the physical activity level, in a quantitative and qualitative approach, and viceversa (**energetic balance**).
- Steamed vegetables offer better nutritional and even organoleptic value; and well-cooked eggs are more secured with essential available proteins (**healthy cooking techniques**).
- Water is essential for the maintenance and development of life, so its daily consumption is extremely important. The recommended intake of **water** is one to two liters of water per day. However, its consumption *at libitum* or according to your needs of physical and intellectual activity will be the most appropriate.

B. **SECOND LEVEL OF THE PYRAMID:**

1. **Whole grain cereals**; the energy intake from this food group should be adjusted according to the physical activity level (**approximately 2 to 4 servings per day**).
2. **Fruits**; daily recommendation. Three or more servings or pieces of varied fruit per day (**at least 3 servings per day**).
3. **Vegetables**; daily recommendation. Incorporate at least two servings of vegetables every day. One of the servings in raw format with varieties of different colors (**at least 2 servings per day**).
4. **Oils and fats**; extra virgin olive oil from any of the many varieties of olives is especially recommended (**at least 1 to 2 tablespoons, or at least 10g per day**).
5. **Meat**; lean meat is part of the traditional Mediterranean Diet and a good choice as a source of protein and other nutrients (**1 to 2 servings, or 100 to 300g, per week**).
6. **Fish and shellfish**; prioritize the consumption of sustainable seasonal fishing. Use the sizes, quantities and culinary preparations that ensure the safety of the product. Eat fish at least two or three times a week (**1 to 2 servings, or 100 to 300g, per week**).
7. **Legumes**; two to three, or more, servings per week is recommended with culinary techniques that improve their digestibility and nutritional value (**at least 2 to 3 servings, or at least 150g per week**).
8. **Nuts and seeds**; prioritize the consumption of local varieties in natural format, or little manipulated without salt or added sugars. The recommended intake is at least four servings per week, each serving of approximately 25 grams (**at least 4 servings of 15 to 25g per week**).
9. **Eggs**; prioritize the ecological varieties, not only for their possible nutritional or organoleptic differentiation, but also for the contribution to humanize the food production chain (**at least 3 units per week**).
10. **Dairy**; good quality dairy products are recommended, two to four servings per day, prioritizing low-fat and without added sugar formats (**2 to 3 servings a day**).

C. **THIRD LEVEL OF THE PYRAMID:**

- The consumption of **red and processed meat**, in all their formats, should be moderate within the framework of a healthy diet.
- The consumption of **fat spreads,** of all kinds, should be moderate and only occasionally, with a preference for unsalted butter.
- Moderate the consumption of **sugar and sugary products**. Do not to exceed 10% of the daily energy intake from added sugars.
- Moderate the consumption of **salt and products with a high concentration of structural or added salt**. Do not exceed the amount of 6g of total salt (sodium chloride) per day.
- An optional, occasional and moderate consumption of industrial **pastries, cakes, sweets, sugary drinks, ice cream and candies** is recommended, prioritizing the consumption of pastries made at home using traditional methods and ingredients.
- Moderate or avoid the consumption of **alcoholic beverages**. The consumption of fermented beverages of low alcohol content and good quality can be assumed in quantities not exceeding two glasses of wine per day in men and one glass per day in women, always referring to adulthood. Consult the research staff in case of chronic diseases or consumption of medicines.

END OF THE GENERAL NUTRITIONAL ADVICE WITH DIETARY RECOMMENDATIONS FOR A HEALTHY LIFESTYLE

THANK YOU VERY MUCH

REFERENCES:

1. Aranceta-Bartrina J, Partearroyo T, Lopez-Sobaler AM, Ortega RM, Varela-Moreiras G, Serra-Majem L, et al. Updating the Food-Based Dietary Guidelines for the Spanish Population: The Spanish Society of Community Nutrition (SENC) Proposal. Nutrients. 2019;11(11).

HOW TO CITE THIS DOCUMENT:

Pantoja-Arévalo L, Gesteiro E, Pérez-Ruiz M, *et al*. The multifactorial approach and the Food-Allergen Specific Substitutive Diet as a tool to manage and ameliorate adverse reactions to foodstuffs in adulthood: Study Protocol for a Randomized Controlled Trial. The ALASKA study. Supplementary Information. *Trials*. 2024.

ANNEX II. INSTRUCTIONS OF THE FOOD-ALLERGEN SPECIFIC SUBSTITUTIVE DIET (FASSD)

INSTRUCTIONS:

Use the following recommendations during the dietary treatment of this study (5 to 6 months). Do not follow these instructions on your own or without the supervision of the investigators of this study.

If you have any questions regarding these recommendations, please contact the research staff at l.pantoja@upm.es, marcela.gonzalez.gross@upm.es, or gi.imfine@upm.es.

1. If you have resulted equal to or greater than level 3 for any of the following foods, follow the substitution directions detailed below.
    - Replace the food and/or beverages to which you have resulted equal to or greater than level 3 and any products that include this foodstuffs as part of their ingredient list (e.g. if you resulted soybean at level equal to or greater than 3; then replace also: soy lecithin, fresh soybeans, soy sauce, tofu, soy vegetable drink, etc.) with any of the substitute foodstuffs described in columns C and D of the Table I.
    - Maintain your usual diet respecting the number of food and/or beverages that you usually have, your consumption preferences, favorite foods, except for making the change or substitution for the foodstuffs to which you resulted equal to or greater than level 3.
    - Maintain your usual physical activity and sport habits during the time of this present dietary treatment (5 to 6 months).
    - When reviewing the list of substitutive foods, make sure you do not have a reaction equal to or greater than level 3 to the foods described in column D.

2. Description of Table I.
    - The following table contains the list of substitutive food and/or beverages in **alphabetical order**.
    - The following table contains the list of substitutive food and/or beverages based on the **food-allergen panels in type I and type II food allergy** for the detection of IgE and IgG4 Ab reactions in human serum samples of AESKUBLOTS® (Aesku.Diagnostics GmbH, Wendelsheim, Germany).
    - The following table contains the list of substitutive food and/or beverages considering mainly their **raw form** (unless otherwise specified).
    - The following table contains the list of substitutive food and/or beverages considering the predominant vitamin and mineral of each raw food according to the Spanish Database of Food Composition BEDCA [(1)]. Particular attention has been paid to the following vitamins and minerals: **Niacin, Mg, K, P, Ca, Zn, B12, Folate, Fe and fiber**.
    - The following table contains the list of substitutive food and/or beverages, which substitute the original measured positive foodstuff (foodstuffs with equal to or greater than level 3 result) considering its nutritional content. Substitutive foodstuffs **do not** always represent a **gastronomic** substitution.
    - Foodstuffs of **Column A** have been **omitted** in Columns C and D, when possible.

POLITÉCNICA

INEF

Table 1 Substitutive foodstuffs according to specific nutrients based on the clinical results of food-specific IgE & IgG4 antibody reactions

	A	B	C	D
	**FOOD AND/OR BEVERAGES**	**NUTRIENT** *cantidad por 100 g de porción comestible*	**SUBSTITUTIVE FOOD AND/OR BEVERAGES**	**NUTRIENTS OF SUBSTITUTIVE FOOD AND/OR BEVERAGES** *cantidad por 100 g de porción comestible*
1	**ALMOND** *Prunus dulcis*	NIACIN (5.3mg)<br>FOLATE (70ug)**<br>POTASSIUM (767.3mg)<br>MAGNESIUM (258.1mg)**<br>PHOSPHORUS (524.9mg)**	**PINE NUTS + FRIED BEANS***, PINE NUTS + ROASTED CHESTNUTS*, PINE NUTS + COCONUT	**PINE NUTS**<br>NIACIN (6.9mg)<br>FOLATE (58ug)**<br>POTASSIUM (780mg)<br>MAGNESIUM (270mg)**<br>PHOSPHORUS (650mg)**<br>**+ FRIED BEANS***<br>FOLATE (78ug)**
2	**AMARANTH** *Amaranthus*	NIACIN (1.8mg)<br>FOLATE (158.3ug)**<br>POTASSIUM (980.4mg)	**BROAD BEAN**, SWEET POTATO, CHICKPEA, FLAX SEED, PUMPKIN SEED	**BROAD BEAN**<br>NIACIN (26mg)<br>FOLATE (423ug)**<br>POTASSIUM (1090mg)
3	**ANACARD** *Anacardium occidentale*	NIACIN (2mg)<br>FOLATE (68ug)**<br>POTASSIUM (552mg)<br>MAGNESIUM (267mg)**<br>PHOSPHORUS (373mg)**	**PINE NUT**, FRIED BROAD BEAN*, ROASTED CHESTNUT*, FLAX SEED, PUMPKIN SEED	**PINE NUT**<br>NIACIN (6.9mg)<br>FOLATE (58ug)**<br>POTASSIUM (780mg)<br>MAGNESIUM (270mg)**<br>PHOSPHORUS (650mg)**
4	**APPLE** *Malus pumila*	VITAMIN C (3mg)<br>POTASSIUM (99mg)	**WATERMELON**, PLUM, PERSIMMON, CHERRY, CUSTARD APPLE	**WATERMELON**<br>VITAMIN C (3mg)<br>POTASSIUM (99mg)
5	**BAKER'S YEAST** *Saccharomyces cerevisiae*	FOLATE (23.4ug)***<br>POTASSIUM (9.6mg)***	**DATE**, COCONUT, POWDER CINNAMON*, CARDO, BROAD BEAN	**DATE**<br>FOLATE (28ug)<br>POTASSIUM (677mg)
6	**BANANA** *Musa paradisiaca*	VITAMIN C (10mg)<br>POTASSIUM (350mg)	**CUSTARD APPLE**, AVOCADO, COCONUT, DATE, RAISIN	**CUSTARD APPLE**<br>VITAMIN C (18mg)<br>POTASSIUM (382mg)
7	**BARLEY** *Hordeum vulgare*	NIACIN (7.8mg)<br>POTASSIUM (560mg)<br>PHOSPHORUS (380mg)**	**PUMPKIN SEED**, CHICKPEA, BROAD BEAN, MUSHROOM, MILLET +FLAXSEED	**PUMPKIN SEED**<br>NIACIN (11.4mg)<br>POTASSIUM (809mg)<br>PHOSPHORUS (1233mg)**
8	**BLACK PEPPER** *Piper nigrum*	VITAMIN C (21mg)<br>VITAMIN A (19ug)**<br>POTASSIUM (1259mg)<br>MAGNESIUM (194mg)**	**DRIED OREGANO* + FRESH PARSLEY**, DRIED DILL* + FRESH PARSLEY, ROGROUPER FISH + DRIED THYME*, CUMIN + FRESH PARSLEY	**DRIED OREGANO***<br>VITAMIN A (690ug)**<br>POTASSIUM (1670mg)<br>MAGNESIUM (270mg)**<br>**+ FRESH PARSLEY**<br>VITAMIN C (190mg)
9	**BROCCOLI** *Brassica oleracea*	VITAMIN C (110mg),<br>POTASSIUM (370mg)	**RED PEPPER+ EGGPLANT**, BRUSSELS SPROUT, FRESH PARSLEY, KALE, FRESH CHIVES (+ COL BLANCA)	**RED PEPPER**<br>VITAMIN C (152mg)<br>POTASSIUM (155mg)<br>**+ EGGPLANT**<br>POTASSIUM (262mg)
10	**BUCKWHEAT** *Fagopyrum esculentum, **does not** contain gluten*	NIACIN (12.6mg)***<br>POTASSIUM (826.3mg)***<br>PHOSPHORUS (623.3mg)** ***	**PUMPKIN SEED + MILLET**, CHICKPEA, BROAD BEAN, MUSHROOM, PUMPKIN SEED +FLAXSEED	**PUMPKIN SEED**<br>NIACIN (11.4mg)<br>POTASSIUM (809mg)<br>PHOSPHORUS (1233mg)**<br>**+ MILLET**<br>NIACIN (4.7mg)<br>POTASSIUM (195mg)

11	**CABBAGE**<br>*Brasica oleracea*	VITAMIN C (49mg)<br>POTASSIUM (270mg)	**RED CABBAGE + RED PEPPER**, FRESH CHIVES, WATERCRESS, KALE, BRUSSELS SPROUT, + ENDIVE	**RED CABBAGE**<br>VITAMIN C (55mg)<br>POTASSIUM (250mg)<br>**+ RED PEPPER**<br>POTASSIUM (155mg)
12	**CARROT**<br>*Daucus carota sativus*	VITAMIN A (1346ug),<br>POTASSIUM (286mg)	**TURNIP GREENS+ SWEET POTATO,** PUMPKIN, SWEET POTATO, ARUGULA, SPINACH, FRESH CHIVES (+ ARUGULA)	**TURNIP GREENS**<br>VITAMIN A (1000ug)<br>**+ SWEET POTATO**<br>VITAMIN A (666.6ug)<br>POTASSIUM (300mg)
13	**CELERY**<br>*Apium graveolens*	VITAMIN A (95ug),<br>POTASSIUM (305mg)	**CHANTERELLE,** KALE, SWISS CHARD, LAMB'S LETTUCE, WATERCRESS	**CHANTERELLE**<br>VITAMIN A (216ug)<br>POTASSIUM (310mg)
14	**CHICKEN**<br>*gallus gallus*	NIACIN (9.9mg)<br>PHOSPHORUS (248mg)	**SARDINE IN OIL***, MACKEREL FISH, HARE, PUMPKIN SEED, PARTRIDGE	**SARDINE IN OIL***<br>NIACIN (12.6mg)<br>PHOSPHORUS (430mg)
15	**COCOA**<br>*Theobroma cacao, sugared powder*	POTASSIUM (400mg)<br>PHOSPHORUS (332mg)**	**POWDER CEREAL"EKO"* + COCONUT VEGETABLE DRINK* + BROWN SUGAR***, INFUSION+ PALMETTOS*, INFUSION+ PICKLES IN VINEGAR*	**POWDER CEREAL"EKO"***<br>PHOSPHORUS (332mg)**<br>**+ COCONUT VEGETABLE DRINK***<br>POTASSIUM (220mg)<br>**+ BROWN SUGAR***<br>POTASSIUM (320mg)
16	**COD**<br>*Gadus morhua*	NIACIN (2.4mg)<br>POTASSIUM (340mg)	**SQUID + PRESERVED COCKLES***, BOILED EEL*, BROAD BEAN, RED BREAM FISH, FLAXSEED	**SQUID**<br>NIACIN (2.8mg)<br>POTASSIUM (316mg)<br>**+ PRESERVED COCKLES***<br>POTASSIUM (43mg)
17	**COFFEE**<br>*Coffea*	*SOLUBLE POWDER*<br>NIACIN (24.9mg)<br>POTASSIUM (4000mg)<br><br>*SOLUBILIZED/ PREPARED FOODSTUFF*<br>NIACIN (5.2mg)<br>POTASSIUM (115mg)	*SOLUBLE POWDER*<br>**POWDER CHICORY"CAFÉ" * *** + COCONUT VEGETABLE DRINK* + BROWN SUGAR* + BROAD BEAN,** INFUSION+ PALMETTOS*, INFUSION+ PICKLES IN VINEGAR*<br><br>*SOLUBILIZED/ PREPARED FOODSTUFF*<br>**COCONUT VEGETABLE DRINK*+BROAD BEAN**	*SOLUBLE POWDER*<br>**POWDER CHICORY"CAFÉ" * *****<br>POTASSIUM (84.3mg)**<br>**+ COCONUT VEGETABLE DRINK***<br>POTASSIUM (220mg)<br>**+ BROWN SUGAR***<br>POTASSIUM (320mg)<br>**+ BROAD BEAN**<br>NIACIN (26mg)<br><br>*SOLUBILIZED/ PREPARED FOODSTUFF*<br>**+ COCONUT VEGETABLE DRINK***<br>POTASSIUM (220mg)<br>**+ BROAD BEAN**<br>NIACIN (26mg)
18	**CORN**<br>*Zea mays*	FOLATE (30ug)<br>POTASSIUM (330mg)<br>PHOSPHORUS (256mg)**	**FLAXSEED**, BROAD BEAN, PINE NUT, MILLET, CHICKPEA	**FLAXSEED**<br>FOLATE (87ug)<br>POTASSIUM (813mg)<br>PHOSPHORUS (642mg)**
19	**COW**<br>*Bos taurus*	NIACIN (6.3mg)<br>POTASSIUM (290mg)	**BASS**, PLAICE, NECORA CRAB, CRAYFISH, RED DEER, VIEIRA	**BASS**<br>NIACIN (6.7mg)<br>POTASSIUM (340mg)
20	**COW'S MILK***<br>*whole milk, contains casein*	POTASSIUM (290mg)<br>CALCIUM (124 mg)**<br>PHOSPHORUS (92mg)**	**POWDER CEREAL"EKO"* + COCONUT VEGETABLE DRINK* + BROWN SUGAR***, WHITE BEAN, BARNACLE, COCKLES, CHIRLA SHELL, WHELKS O BOILED SNAIL*	**POWDER CEREAL"EKO"**<br>PHOSPHORUS (332mg)**<br>**+ COCONUT VEGETABLE DRINK, CALCIUM FORTIFIED* *****<br>POTASSIUM (220mg)<br>CALCIUM (120-210 mg)**<br>**+ BROWN SUGAR***<br>POTASSIUM (320mg)

21	**EGG WHITE**<br>*Gallus gallus, chicken egg white*	RIBOFLAVIN (0.4mg)<br>POTASSIUM (142mg)	**CHICKPEA + PAPRIKA,** BROAD BEAN, PINTO BEAN, WHITE BEAN, ARUGULA	**CHICKPEA**<br>POTASSIUM (1000mg)<br>**+ PAPRIKA**<br>RIBOFLAVIN (1.7mg)
22	**EGG YOLK**<br>*Gallus gallus, chicken egg yolk*	RIBOFLAVIN (0.50mg)<br>PHOSPHORUS (520mg)	**FLAXSEED + DRIED OREGANO***, DRIED DILL*, PINE NUT, PUMPKIN SEED, SARDINE	**FLAXSEED**<br>RIBOFLAVIN (0.16mg)<br>PHOSPHORUS (642mg)<br>**+ DRIED OREGANO***<br>RIBOFLAVIN (0.53mg)
23	**GARLIC**<br>*Allium sativum*	VITAMIN C (14mg),<br>POTASSIUM (446mg)	**ARUGULA + CUCUMBER**, AVOCADO, ZUCCHINI, TURNIP, PUMPKIN + RED PEPPER	**ARUGULA**<br>VITAMIN C (15mg),<br>POTASSIUM (369mg)<br>**+ CUCUMBER**<br>POTASSIUM (150mg)
24	**GINGER**<br>*Zingiber officinale*	VITAMIN C (4mg)<br>VITAMIN A (5.8mg)**<br>POTASSIUM (330mg)	**CURRY SALSA***, TABASCO SALSA*, GREEN SPICY PEPPER, RED, PAPRIKA*, CHILLI POWDER PEPPER*	**CURRY SALSA***<br>VITAMIN C (11.4mg)<br>VITAMIN A (100mg)**<br>POTASSIUM (1550mg)
25	**GOAT'S MILK***<br>*whole milk, contains casein*	POTASSIUM (185mg)<br>CALCIUM (120 mg)**<br>PHOSPHORUS (103mg)**	**POWDER CEREAL"EKO"* + COCONUT VEGETABLE DRINK, CALCIUM FORTIFIED* + BROWN SUGAR**, VIEIRA, WHITE BEAN, PINTO BEAN, BARNACLE, COCKLES	**POWDER CEREAL"EKO"**<br>PHOSPHORUS (332mg)**<br>**+ COCONUT VEGETABLE DRINK, CALCIUM FORTIFIED*** ***<br>POTASSIUM (220mg)<br>CALCIUM (120-210 mg)**<br>**+ BROWN SUGAR***<br>POTASSIUM (320mg)
26	**GRAPE**<br>*Vitis vinifera l*	VITAMIN C (4mg)<br>POTASSIUM (250mg)	**CHERRY**, WATERMELON, PLUM, QUINCE, CUSTARD APPLE	**CHERRY**<br>VITAMIN C (8mg)<br>POTASSIUM (255mg)
27	**GREEN BEANS**<br>*Phaseolus vulgaris*	FOLATE (66ug)<br>POTASSIUM (243mg)	**SPINACHS,** LUPINS, CHARD + PUMPKIN, MUSHROOMS + BRUSSELS SPROUT	**SPINACH**<br>FOLATE (143ug)<br>POTASSIUM (380mg)
28	**GREEN PEA**<br>*Pisum sativum*	NIACIN (5.2mg)<br>FOLATE (42ug)**<br>POTASSIUM (900mg)<br>PHOSPHORUS (330mg)**	**WHITE BEAN**, PINTO BEAN, BROAD BEAN + CHANTERELLE, BRUSSELS SPROUT + MUSHROOMS	**WHITE BEAN**<br>NIACIN (5.3mg)<br>FOLATE (388ug)**<br>POTASSIUM (1337mg)<br>PHOSPHORUS (426mg)**
29	**HAZELNUT**<br>*Corylus avellana*	NIACIN (3.4mg)<br>FOLATE (71ug)**<br>POTASSIUM (636mg)<br>MAGNESIUM (156mg)**<br>PHOSPHORUS (333mg)**	**PINE NUT + FRIED BROAD BEAN***, PINE NUT + ROASTED CHESTNUT*, PINE NUT + COCONUT	**PINE NUT**<br>NIACIN (6.9mg)<br>FOLATE (58ug)**<br>POTASSIUM (780mg)<br>MAGNESIUM (270mg)**<br>PHOSPHORUS (650mg)**<br>**+ FRIED BROAD BEAN***<br>FOLATE (78ug)**
30	**KIWI**<br>*Actinidia chinensis*	VITAMIN C (59mg)<br>POTASSIUM (290mg)	**GUAVA**, LICHI, PAPAYA, BLACKCURRANT, LIME	**GUAVA**<br>VITAMIN C (273mg)<br>POTASSIUM (290mg)
31	**LAMB**<br>*Ovis orientalis aries*	NIACIN (3.3mg)<br>POTASSIUM (301.5mg)	**HALIBUT FISH**, RED BREAM FISH, WATERCRESS, PUMPKIN, RED BREAM FISH, CHANTERELLE	**HALIBUT FISH**<br>NIACIN (6.28mg)<br>POTASSIUM (309mg)
32	**LEEK**<br>*Allium porrum*	VITAMIN C (18mg)<br>POTASSIUM (256mg)	**ZUCCHINI + TURNIP**, GREEN ASPARAGUS, RADISH, BROAD BEAN	**ZUCCHINI**<br>VITAMIN C (20mg)<br>POTASSIUM (230mg)<br>**+ TURNIP**<br>POTASSIUM (238mg)

33	**LEMON**<br>*Citrus limon, fresh juice*	VITAMIN C (37mg)<br>POTASSIUM (123mg)	**NECTARINE**, GRAPEFRUIT, PARAGUAYA PEACH, LIME, LICHI	**NECTARINE**<br>VITAMIN C (37mg)<br>POTASSIUM (170mg)
34	**MANGO**<br>*Mangifera indica*	VITAMIN C (44mg)<br>POTASSIUM (150mg)	**LICHI**, PAPAYA, BLACKCURRANT, GUAVA, LIME	**LICHI**<br>VITAMIN C (60mg)<br>POTASSIUM (156mg)
35	**MUSTARD**<br>*Sinapis alba*	VITAMIN C (75mg)<br>FOLATE (18ug)**<br>SODIUM (2245mg)<br>PHOSPHORUS (202mg)**	**CHILLI PEPPER***, FRESH PARSLEY + SAFFRON, FRESH PARSLEY + CUMIN	**CHILLI PEPPER***<br>VITAMIN C (143.7mg)<br>FOLATE (23ug)**<br>SODIUM (9mg)<br>PHOSPHORUS (43mg)**
36	**NUT**<br>*Juglans regia*	NIACIN (3.5mg)<br>FOLATE (66ug)**<br>POTASSIUM (690mg)<br>MAGNESIUM (140mg)**<br>PHOSPHORUS (304mg)**	**PINE NUT + FRIED BROAD BEAN***, PINE NUT + ROASTED CHESTNUT*, PINE NUT + COCONUT	**PINE NUT**<br>NIACIN (6.9mg)<br>FOLATE (58ug)**<br>POTASSIUM (780mg)<br>MAGNESIUM (270mg)**<br>PHOSPHORUS (650mg)**<br>**+ FRIED BROAD BEAN***<br>FOLATE (78ug)**
37	**OAT**<br>*Avena sativa*	FOLATE (56ug)<br>POTASSIUM (429mg)<br>PHOSPHORUS (523mg)**	**FLAXSEED**, BROAD BEAN, PINE NUT, MILLET, CHICKPEA	**FLAXSEED**<br>FOLATE (87ug)<br>POTASSIUM (813mg)<br>PHOSPHORUS (642mg)**
38	**OCTOPUS**<br>*Octopoda*	POTASSIUM (230mg)<br>SODIUM (363mg)**	**SARDINE IN OIL***, CRAYFISH, NECORA CRAB, CANGREJO, SPIDER CRAB	**SARDINE IN OIL***<br>POTASSIUM (388mg)<br>SODIUM (366mg)**
39	**ONION**<br>*Allium cepa*	VITAMIN C (6.9mg)<br>POTASSIUM (162mg)	**ENDIVE**, ESCAROLE, PUMPKIN, ARUGULA, TURNIP	**ENDIVE**<br>VITAMIN C (10mg)<br>POTASSIUM (205mg)
40	**ORANGE**<br>*Citrus aurantium l*	VITAMIN C (50mg)<br>POTASSIUM (200mg)	**BLACKCURRANT,** PAPAYA, LICHI, BLACKCURRANT, GUAVA	**BLACKCURRANT**<br>VITAMIN C (159.6mg)<br>POTASSIUM (341.9mg)
41	**PEACH**<br>*Prunus persica*	VITAMIN C (8mg)<br>POTASSIUM (260mg)	**MELON**, QUINCE, CUSTARD APPLE, POMEGRANATE, BLUEBERRY,	**MELON**<br>VITAMIN C (25mg)<br>POTASSIUM (320mg)
42	**PEANUT**<br>*Arachis hypogaea*	NIACIN (18.4mg)<br>FOLATE (110ug)**<br>POTASSIUM (670mg)<br>MAGNESIUM (210mg)**<br>PHOSPHORUS (430mg)**	**PINE NUT + FRIED BROAD BEAN* + PUMPKIN SEED**, PINE NUT + ROASTED CHESTNUT* + FLAXSEED, PINE NUT + COCONUT + PUMPKIN SEED	**PINE NUT**<br>NIACIN (6.9mg)<br>FOLATE (58ug)**<br>POTASSIUM (780mg)<br>MAGNESIUM (270mg)**<br>PHOSPHORUS (650mg)**<br>**+ FRIED BROAD BEAN***<br>NIACIN (4.1mg)<br>FOLATE (78ug)**<br>**+ PUMPKIN SEED**<br>NIACIN (11.4mg)
43	**PINEAPPLE**<br>*Ananas comosus*	VITAMIN C (20mg)<br>POTASSIUM (250mg)	**POMEGRANATE + BLUEBERRY**, RASPBERRY, NECTARINE, PARAGUAYA PEACH	**POMEGRANATE**<br>VITAMIN C (20mg)<br>POTASSIUM (247mg)<br>**+ BLUEBERRY**<br>POTASSIUM (78mg)
44	**PORK**<br>*Sus scrofa, lean meat*	NIACIN (5.1mg)<br>POTASSIUM (435mg)	**TRUCHA + EGGPLANT**, BROAD BEAN, BORRAJA, MUSHROOM, VIEIRA	**TRUCHA**<br>NIACIN (5.1mg)<br>POTASSIUM (250mg)<br>**+ EGGPLANT**<br>POTASSIUM (262mg)

45	**POTATO**<br>*Solanum tuberosum*	NIACIN (1.15mg)<br>POTASSIUM (525mg)	**SWEET POTATO + AVOCADO**, BEET + AVOCADO, GREEN ASPARAGUS + MUSHROOM	**SWEET POTATO**<br>NIACIN (0.8mg)<br>POTASSIUM (300mg)<br>**+ AVOCADO**<br>NIACIN (1.5mg)<br>POTASSIUM (400mg)
46	**QUINOA**<br>*Chenopodium quinoa*	NIACIN (2.9mg)<br>POTASSIUM (780mg)<br>PHOSPHORUS (230mg)**	**PUMPKIN SEED**, CHICKPEA, BROAD BEAN, MUSHROOM, MILLET +FLAXSEED	**PUMPKIN SEED**<br>NIACIN (11.4mg)<br>POTASSIUM (809mg)<br>PHOSPHORUS (1233mg)**
47	**RICE**<br>*Oryza sativa*	NIACIN (2.4mg)<br>FOLATE (20ug)**<br>POTASSIUM (110mg)	**BROAD BEAN**, SWEET POTATO, CHICKPEA, FLAXSEED, PUMPKIN SEED, PUMPKIN SEED, ETC.	**BROAD BEAN**<br>NIACIN (26mg)<br>FOLATE (423ug)**<br>POTASSIUM (1090mg)
48	**RYE**<br>*Secale cereale, contains gluten*	NIACIN (4.3mg)<br>POTASSIUM (264mg)<br>PHOSPHORUS (374mg)**	**PUMPKIN SEED**, CHICKPEA, BROAD BEAN, MUSHROOM, MILLET +FLAXSEED	**PUMPKIN SEED**<br>NIACIN (11.4mg)<br>POTASSIUM (809mg)<br>PHOSPHORUS (1233mg)**
49	**SALMON**<br>*Salmo salar*	NIACIN (10.4mg)<br>POTASSIUM (310mg)	**MACKEREL FISH**, PUMPKIN SEED, HARE, PARTRIDGE, SARDINE IN OIL*	**MACKEREL FISH**<br>NIACIN (12.5mg)<br>POTASSIUM (386mg)
50	**SESAME**<br>*Sesamum indicum*	NIACIN (4.5mg)<br>FOLATE (96ug)**<br>POTASSIUM (450mg)<br>CALCIUM (150mg)**<br>MAGNESIUM (350mg)**<br>PHOSPHORUS (620mg)**	**FLAXSEED + GREEN ASPARAGUS,** CAVIAR, SARDINE IN OIL*, SARPA, ANCHOVIES IN OIL* + FRESH PARSLEY	**FLAXSEED**<br>NIACIN (3.1mg)<br>FOLATE (87ug)**<br>POTASSIUM (813mg)<br>CALCIUM (255mg)**<br>MAGNESIUM (392mg)**<br>PHOSPHORUS (642mg)**<br>**+ GREEN ASPARAGUS**<br>NIACIN (1.5mg)<br>FOLATE (113ug)**
51	**SHEEP'S MILK***<br>*whole milk, contains casein*	POTASSIUM (146mg)<br>CALCIUM (183 mg)**<br>PHOSPHORUS (141mg)**	**POWDER CEREAL"EKO"* + COCONUT VEGETABLE DRINK* + BROWN SUGAR***, ANCHOVIES IN OIL*, FLAXSEED, SARDINE IN OIL*, PINTO BEAN	**POWDER CEREAL"EKO"**<br>PHOSPHORUS (332mg)**<br>**+ COCONUT VEGETABLE DRINK, CALCIUM FORTIFIED* *****<br>POTASSIUM (220mg)<br>CALCIUM (120-210 mg)**<br>**+ BROWN SUGAR***<br>POTASSIUM (320mg)
52	**SHRIMP**<br>*Parapenaeus longirostris*	SODIUM (914.3mg)<br>PHOSPHORUS (323.8mg)**	**PRESERVED COCKLES* + MONKFISH**, PICKLES IN VINEGAR*, CAPER, ANCHOVIES IN OIL*, WHELKS O BOILED SNAIL	**PRESERVED COCKLES***<br>SODIUM (960mg)<br>PHOSPHORUS (30mg)**<br>**+ MONKFISH**<br>PHOSPHORUS (330mg)
53	**SOYBEAN**<br>*Glycine max*	POTASSIUM (620mg)<br>CALCIUM (197mg)**	**FLAXSEED,** CAVIAR, SARDINE IN OIL*, SARPA, ANCHOVIES IN OIL* +FRESH PARSLEY	**FLAXSEED**<br>POTASSIUM (813mg)<br>CALCIUM (255mg)**
54	**SPELT OR SPELT WHEAT**<br>*Triticum spelta*	NIACIN (11.9mg)***<br>POTASSIUM (675.1mg)***<br>PHOSPHORUS (697.7mg)** ***	**PUMPKIN SEED**, CHICKPEA, BROAD BEAN, MUSHROOM, MILLET +FLAXSEED	**PUMPKIN SEED**<br>NIACIN (11.4mg)<br>POTASSIUM (809mg)<br>PHOSPHORUS (1233mg)**
55	**SQUID**<br>*Loligo vulgaris*	NIACIN (2.8mg)<br>POTASSIUM (316mg)	**PRESERVED MUSSEL IN WATER***, RED BREAM FISH, BIOLED OCTOPUS*, COOKED MUSHROOM*, PERCH	**PRESERVED MUSSEL IN WATER***<br>NIACIN (3mg)<br>POTASSIUM (320mg)
56	**STRAWBERRY**<br>*Fragaria vesca*	VITAMIN C (60mg)<br>POTASSIUM (190mg)	**PAPAYA,** BLACKCURRANT, GUAVA (WITHOUT FRUIT'S PEEL), LICHI, LIME	**PAPAYA**<br>VITAMIN C (64mg)<br>POTASSIUM (214mg)

**57**	**TOMATO**<br>*Lycopersicum esculentum*	VITAMIN A (82ug)<br>POTASSIUM (236mg)	**KALE**, CHARD, CHANTERELLE, LAMB'S LETTUCE, WATERCRESS	**KALE**<br>VITAMIN A (165.83ug),<br>POTASSIUM (320mg)
**58**	**TROUT**<br>*Salmo trutta*	POTASSIUM (250mg)<br>PHOSPHORUS (208mg)**	**RED BREAM FISH**, RED DEER, BLEAK, GROUPER FISH, POUT	**RED BREAM FISH**<br>POTASSIUM (310mg)<br>PHOSPHORUS (210mg)**
**59**	**TUNA**<br>*Thunnus thynnus*	NIACIN (12.8mg)<br>POTASSIUM (400mg)	**ALBACORE IN OIL* + SOLE FISH**, BROAD BEAN, SARDINE IN OIL*, MACKEREL FISH, PARTRIDGE	**ALBACORE IN OIL***<br>NIACIN (13.5mg)<br>POTASSIUM (267mg)<br>**+ SOLE FISH**<br>POTASSIUM (230mg)
**60**	**TURKEY**<br>*Meleagris gallopavo*	NIACIN (10.6mg)<br>POTASSIUM (247mg)	**ALBACORE IN OIL***, PARTRIDGE, MACKEREL FISH, SARDINE IN OIL*, BROAD BEAN	**ALBACORE IN OIL***<br>NIACIN (13.5mg)<br>POTASSIUM (267mg)
**61**	**WHEAT**<br>*Triticum, contains gluten*	NIACIN (8.2mg)<br>POTASSIUM (421mg)<br>PHOSPHORUS (344.4mg)**	**PUMPKIN SEED**, CHICKPEA, BROAD BEAN, MUSHROOM, MILLET +FLAXSEED	**PUMPKIN SEED**<br>NIACIN (11.4mg)<br>POTASSIUM (809mg)<br>PHOSPHORUS (1233mg)**

*Processed food (not raw).
** >2 nutrients have been considered due to their importance in the allergenic food.
***USDA (2).
**** NCCDB (3).

END OF THE INSTRUCTIONS OF THE FOOD-ALLERGEN SPECIFIC SUBSTITUTIVE DIET (FASSD)

THANK YOU VERY MUCH

REFERENCES:

1. AESAN/BEDCA. Spanish Food Composition Database BEDCA. In: RedBEDCA/AESAN, editor. https://www.bedca.net2010.

2. U.S. Department of Agriculture Agricultural Research Service NDL. USDA National Nutrient Database for Standard Reference. In: USDA, editor. 26 ed. http://www.ars.usda.gov/ba/bhnrc/ndl2013.

3. Harnack L. Nutrition Data System for Research (NDSR) and The University of Minnesota Nutrition Coordinating Center (NCC) Food and Nutrient Database as the source of food composition information. In: Gellman MD, Turner JR, editors. Encyclopedia of Behavioral Medicine. New York, NY: Springer New York; 2013. p. 1348–50.

HOW TO CITE THIS DOCUMENT:

Pantoja-Arévalo L, Gesteiro E, Pérez-Ruiz M, *et al*. The multifactorial approach and the Food-Allergen Specific Substitutive Diet as a tool to manage and ameliorate adverse reactions to foodstuffs in adulthood: Study Protocol for a Randomized Controlled Trial. The ALASKA study. Supplementary Information. *Trials*. 2024.

ANNEX III. 24HDRI: 24-HOUR DIETARY RECALL INTERVIEW

24-HOUR DIETARY RECALL INTERVIEW
FOOD AND/OR BEVERAGES CONSUMPTION INTERVIEW
OF THE PAST 24 HOURS (THE DAY BEFORE) TO THE SUBJECT OF STUDY

PROYECT NUMBER:______________________ **INTERVIEW DATE:**_____ /_____ / 202__

FOLLOW-UP PROYECT CODE:______________________

PARTICIPANT CODE: ______________________ (If you haven't receive your participant code, please contact the research staff at l.pantoja@upm.es, marcela.gonzalez.gross@upm.es, or gi.imfine@upm.es and request the unique participant code that you will use throughout the entire study as identification. If possible, memorize this code)

INSTRUCTIONS:

First, we would like to thank you for participating in this study. We will be able to obtain relevant information about the diet you are currently following, and to contribute to the development of the scientific knowledge.

During this interview, it is very important to consider all foodstuffs, **food and/or beverages**, without forgetting those that you have taken **between meals** such as: snacks, fruits, pastries, candies, chewing gums or others, as well as those **ingredients** that are added to meals once prepared: salt, sugar, honey, cocoa, sweetener, etc. Likewise, it is especially important to remember all the consumed **beverages** such as: water, juice, soft drink, smoothie, tea, coffee, infusion or other additional drink taken either at main meals or between meals.

During this interview, you must also describe all **food supplements and/or medication** that you have consumed yesterday or during the past 24 hours (e.g., vitamins, minerals, aminoacids or proteins, fat burners, dietary fibers, diuretics, cleansers, probiotics, prebiotics, antioxidants, collagens, appetite control and suppressants, fat and carbohydrate blockers, hyaluronic acid or any other food supplement).

STUCTURE OF THE RECALL INTERVIEW:

1. In the first column, talk about the **time** of the day at which you **started and finished** consuming the foodstuffs of each section (i.e., breakfast, between meals, lunch, dinner and meal after dinner), as well as, the **place** where you consumed them (e.g., at home, friends' house, parents' house, cafeteria, bar, restaurant, workplace, university, during a trip, outdoor picnic, etc.).
2. In the second column, provide a general description of the corresponding **menu and its food preparation**: fried, roasted, grilled, sautéed, stewed, boiled, steamed, etc. In this column, you must also state whether there is a **partial or total elimination of any ingredient or foodstuff** either because it causes discomfort or due to medical recommendation, such as: salt, milk and dairy products, gluten, eggs, nuts, fish, etc.
3. In the third column, you should detail the **type of foodstuffs** on each of the mentioned meals, offering special attention to:
   - Type of dairy product: butter, margarine, cream, yogurt, milk, cheese, shake or other.
   - Type of yogurt: natural, sweetened, sugared, 0/0.
   - Type of milk: whole, skimmed or semi-skimmed, enriched or fortified (explain the nutrient: omega 3, omega 6, calcium, vitamin D, Fe).
   - Type of cheese: manchego, roquefort, gouda, parmesan, fresh, ricotta or cottage.
   - Type of oil: olive (virgin or extra virgin), sunflower, sesame.
   - Type of bread: wheat, spelled, buckwheat, rye, whole wheat, corn. And if it is homemade, bar or mold.
   - Product Brand: (if applicable and remembered).
4. Finally, in the last column, describe the **consumed amount** of each foodstuff, food and/or beverages, as accurately as possible. You can specify the amount consumed using household measurements (glasses, cups, tablespoons, saucepans, capsules, etc.), or also, if you have the information, in grams or milliliters. Remember the leftovers and exclude them from the final count.

PARTICIPANT CODE: A L

RESEARCHERS: ___________________________
(INITIALS OF THE RESEARCHERS)

GUIDE OF FOOD, BEVERAGES AND FOOD SUPPLEMENTS (EXAMPLES)

Please find below examples of how to describe some food, beverages and food supplements:

- The following guide is alphabetically organized.

**ALCOHOLIC BEVERAGES**	Explain the type of alcoholic beverage: liquor, cognac, whiskey, vodka, gin, rum. Specify the alcohol content, in %, indicated on the label. State the commercial brand, if remembered.
**BEER**	Explain the type of beer: non-alcoholic, malt beer, wheat beer, rice beer. Specify the consumed amount of beer in: can, bottle, cane, jug, glass, etc. State the commercial brand, if remembered.
**BREAD**	Explain whether it is white bread, whole wheat, rye, multigrain, rustic, or any other type of bread. Describe if it is homemade, bar or mold. Specify the consumed amount of bread: number of slices or pieces or, at least, the approximate size of the portions, using the visual guide of portion sizes provided by the investigator (interviewer) of this study (1). State the commercial brand, if remembered.
**COFFEE, TEA AND HERBAL INFUSIONS**	Describe whether it is regular coffee or decaffeinated coffee. Describe the type of coffee: capsule, soluble and instant. Explain the type of tea or herbal infusion: green tea, black tea, chamomile infusion, pennyroyal, etc. Describe the type and amount of consumed sweetener: white sugar, brown sugar, saccharin, honey, stevia, etc.
**EGGS**	Explain the animal of origin: chicken, duck, ostrich, quail, etc. If you know it, specify the origin: industrial, ecological, organic. Describe the weight and size: Super Large (XL: >73g), Large (L: 63-73g), Medium (M: 53-63g) or Small (S: < 53g).
**FISH**	Describe the name, type of fish and the serving size using the visual guide of portion sizes provided by the investigator (interviewer) of this study (1).
**FOOD SUPPLEMENTS**	Describe the type of food supplement: vitamins, minerals, aminoacids or proteins, fat burners, dietary fibers, diuretics, cleansers, probiotics, prebiotics, antioxidants, collagens, appetite control and suppressants, fat and carbohydrate blockers, hyaluronic acid or any other food supplement. Explain the type of presentation: tablets, capsules, drinks, granules, powder. Describe the consumed amount and the frequency of consumption of food supplements: daily, weekly, monthly. State the commercial brand, if remembered. If possible, provide to the interviewer a photocopy of the composition and list of ingredients of the consumed food supplements.
**FRUITS AND VEGETABLES**	Explain the type and size of the portion using the visual guide of portion sizes provided by the investigator (interviewer) of this study (1).
**MEAT**	Explain the animal of origin (pork, beef, rabbit, chicken), the cut (hip, thigh, breast) and the type of preparation (fried, roasted, grilled, sautéed, stewed, boiled, steamed). In the case of chicken, explain whether you eat the skin or not.
**MILK AND DAIRY PRODUCTS**	Explain the animal of origin: cow, goat, sheep, etc. Explain the type of milk or dairy product: whole milk, semi-skimmed milk, skimmed milk; Manchego cheese (cured, semi-cured, tender, aged), Roquefort or blue cheese, gouda cheese, parmesan cheese, ricotta cheese or cottage cheese, fresh cheese; liquid yogurt, Greek yogurt (specify flavor; if sweetened, sugared, 0/0 or natural; and if skimmed, whole). Describe whether the foodstuff is: lactose-free, enriched in calcium, omega 3, omega 6, vitamin D, and the consumed amount in glasses, grams, slices or portions. State the commercial brand, if remembered.
**NON-ALCOHOLIC BEVERAGES**	Explain the type of non-alcoholic beverage: soft drink, fruit juice drink, nectar. Explain the consumed amount in glasses, cups, bottles, etc.
**OILS ANS FATS**	Describe the type of oils and fats: extra virgin olive oil (EVOO), sunflower oil, butter, margarine. Explain the consumed amount in approximate number of tablespoons.
**PASTRIES**	Explain the type: croissant, muffins, cakes, palm trees, churros, porras. Describe the consumed amount in serving size using the visual guide of portion sizes provided by the investigator (interviewer) of this study (1). Describe if it is homemade, bakery or industrial.
**PREPARES MEALS**	Describe the product, indicating the type of packaging (can, sachet, glass jar, frozen bag, frozen carton) and the consumed amount in portion sizes. State the commercial brand, if remembered.
**SALAD**	Describe the composition of the salad: lettuce, tomato, onion, spinach, dressing (salt, pepper, vinegar, extra virgin olive oil, sunflower oil) and the consumed amount in portion sizes.
**SAUCES**	Explain the type of sauce (mayonnaise, ketchup, mustard, pink sauce, soy sauce or hot sauce: chilli, curry) and describe the consumed amount of sauce in tablespoons, ladles, single-dose sachets. State whether they are packaged (industrial), homemade (if homemade, describe their composition). State the commercial brand, if remembered.
**SAUSAGE, COLD CUT AND CURED MEAT**	Describe the type: turkey ham, pork ham, Iberian ham, pork smoked ham, pork sausage, mortadella. Specify the number of slices or grams.
**SOUPS AND MASHED VEGETABLES**	Describe the meal composition and explain the quantity in cups, plates, tablespoons, using the visual guide of portion sizes provided by the investigator (interviewer) of this study (1).
**SPICES**	Explain the type of spice: salt, basil, thyme, cumin, pepper, parsley, oregano, curry, paprika and the consumed amount in tablespoons or teaspoons, using the visual guide of portion sizes provided by the investigator (interviewer) of this study (1).
**WATER**	Specify whether it is bottled, tap, or filtered water. Explain the type of water, mineral, sparkling, still, natural. Explain the consumed amount in number of glasses, glass size or liters, using the visual guide of portion sizes provided by the investigator (interviewer) of this study (1).
**WINE**	Explain the type of wine by type of grape, fermentation grade, year and brand. Explain the consumed amount , using the visual guide of portion sizes provided by the investigator (interviewer) of this study (1). State the commercial brand, if remembered.

PARTICIPANT CODE: A L _ _ _ _

RESEARCHERS: ___________________________

(INITIALS OF THE RESEARCHERS)

24-HOUR DIETARY RECALL INTERVIEW

FOOD AND/OR BEVERAGES CONSUMPTION INTERVIEW

OF THE PAST 24 HOURS (THE DAY BEFORE) TO THE SUBJECT OF STUDY

INTERVIEWER (INITIALS): ______________________________ **INTERVIEW DATE:** _____ /_____ / 20 ___

PARTICIPANT CODE: ____________________________ (If you haven't receive your participant code, please contact the research staff at l.pantoja@upm.es, marcela.gonzalez.gross@upm.es, or gi.imfine@upm.es and request the unique participant code that you will use throughout the entire study as identification. If possible, memorize this code)

SELECT (X) THE DAY OF THE WEEK THAT WAS YESTERDAY:

☐MONDAY ☐TUESDAY ☐WEDNESDAY ☐THURSDAY ☐FRIDAY ☐SATURDAY ☐SUNDAY

**FOOD AND/OR BEVERAGES AND FOOD SUPPLEMENTS**			
**TIME (HH:MM) AND PLACE OF FOODSTUFFS CONSUMPTION**	**MEAL DESCRIPTION**<br>(First dish, second dish, dessert and drink. Specify the cooking type: fried, roasted, grilled, stewed, boiled, steamed. Specify whether there is a partial or total elimination of any ingredient or foodstuff: gluten, milk, nuts, etc.)	**INGREDIENTS, COMPOSITION AND MEAL CHARACTERISTICS**	**QUANTITY (g, ml, units)**<br><br>**OR HOUSEHOLD MEASUREMENT (spoon, handful)**
**BREAKFAST**			
Start time:<br><br>End time:<br><br>Place:			
**MID-MORNING**			
Start time:<br><br>End time:<br><br>Place:			

PARTICIPANT CODE: A L _ _ _ _

RESEARCHERS: ___________________________

(INITIALS OF THE RESEARCHERS)

**TIME (HH:MM) AND PLACE OF FOODSTUFFS CONSUMPTION**	**MEAL DESCRIPTION** (First dish, second dish, dessert and drink. Specify the cooking type: fried, roasted, grilled, stewed, boiled, steamed. Specify whether there is a partial or total elimination of any ingredient or foodstuff: gluten, milk, nuts, etc.)	**INGREDIENTS, COMPOSITION AND MEAL CHARACTERISTICS**	**QUANTITY (g, ml, units) OR HOUSEHOLD MEASUREMENT (spoon, handful)**
**LUNCH**			
Start time:<br>End time:<br>Place:			
**MID-AFTERNOON**			
Start time:<br>End time:<br>Place:			

PARTICIPANT CODE: A L _ _ _ _

RESEARCHERS: ______________________________
(INITIALS OF THE RESEARCHERS)

**TIME (HH:MM) AND PLACE OF FOODSTUFFS CONSUMPTION**	**MEAL DESCRIPTION**<br>(First dish, second dish, dessert and drink. Specify the cooking type: fried, roasted, grilled, stewed, boiled, steamed. Specify whether there is a partial or total elimination of any ingredient or foodstuff: gluten, milk, nuts, etc.)	**INGREDIENTS, COMPOSITION AND MEAL CHARACTERISTICS**	**QUANTITY (g, ml, units)**<br><br>**OR HOUSEHOLD MEASUREMENT (spoon, handful)**
**IN-BETWEEN MEALS**			
Start time:<br><br>End time:<br><br>Place:			
**DINNER**			
Start time:<br><br>End time:<br><br>Place:			
**AFTER DINNER AND/OR OVERNIGHT**			
Start time:<br><br>End time:<br><br>Place:			

TIME (HH:MM)	FOOD SUPPLEMENT AND/OR MEDICATION NAME	MG (CONCENTRATION)	CHARACTERISTICS OF THE FOOD SUPPLEMENT OR MEDICATION (BRAND)	TREATMENT DURATION (30 DAYS, 2 WEEKS)	TIME OF THE DAY WHERE CONSUMED
FOOD SUPPLEMENT AND/OR MEDICATION					
					○ BREAKFAST<br>○ MID-MORNING<br>○ LUNCH<br>○ MID-AFTERNOON<br>○ DINNER<br>○ IN-BETWEEN MEALS<br>○ AFTER DINNER AND/OR OVERNIGHT
					○ BREAKFAST<br>○ MID-MORNING<br>○ LUNCH<br>○ MID-AFTERNOON<br>○ DINNER<br>○ IN-BETWEEN MEALS<br>○ AFTER DINNER AND/OR OVERNIGHT
					○ BREAKFAST<br>○ MID-MORNING<br>○ LUNCH<br>○ MID-AFTERNOON<br>○ DINNER<br>○ IN-BETWEEN MEALS<br>○ AFTER DINNER AND/OR OVERNIGHT
					○ BREAKFAST<br>○ MID-MORNING<br>○ LUNCH<br>○ MID-AFTERNOON<br>○ DINNER<br>○ IN-BETWEEN MEALS<br>○ AFTER DINNER AND/OR OVERNIGHT
					○ BREAKFAST<br>○ MID-MORNING<br>○ LUNCH<br>○ MID-AFTERNOON<br>○ DINNER<br>○ IN-BETWEEN MEALS<br>○ AFTER DINNER AND/OR OVERNIGHT
					○ BREAKFAST<br>○ MID-MORNING<br>○ LUNCH<br>○ MID-AFTERNOON<br>○ DINNER<br>○ IN-BETWEEN MEALS<br>○ AFTER DINNER AND/OR OVERNIGHT

END OF THE 24-HOUR DIETARY RECALL INTERVIEW

THANK YOU VERY MUCH

REFERENCES:

1. Gómez Candela CLK, Viviana; Lourenço Nogueira, Thais. Guía visual de alimentos y raciones. Editores Médicos S.A. (EDIMSA), editor. 2008, 180 p.

2. González-Gross M., Castillo M. J., Moreno L., Nova E., González-Lamuño D., Pérez-Llamas F. et al . Alimentación y valoración del estado nutricional de los adolescentes españoles (Estudio AVENA): Evaluación de riesgos y propuesta de intervención. I. Descripción metodológica del proyecto. Nutr. Hosp. 2003; 18(1): 15-28.

HOW TO CITE THIS DOCUMENT:

Pantoja-Arévalo L, Gesteiro E, Pérez-Ruiz M, *et al*. The multifactorial approach and the Food-Allergen Specific Substitutive Diet as a tool to manage and ameliorate adverse reactions to foodstuffs in adulthood: Study Protocol for a Randomized Controlled Trial. The ALASKA study. Supplementary Information. *Trials*. 2024.

POLITÉCNICA

PARTICIPANT CODE: A L

RESEARCHERS: ________________________

(INITIALS OF THE RESEARCHERS)

INEF

ALASKA Study. ALLERGIES AND FOOD INTOLERANCES IN ADULTS AND ATHLETES

ANNEX IV. DIETARY ADHERENCE QUESTIONNAIRE (DAQ)

INSTRUCTIONS:

- Complete the following questions of the Dietary Adherence Questionnaire (DAQ).
- The questions in this questionnaire refer to the compliance of the recommended dietary instructions (provided during your previous visit) during the **last month**.
- Answer and carefully read each of the following questions.
- The grey boxes are reserved to be fulfilled by the researcher (s). Do not complete the grey boxes.
- This questionnaire takes approximately 6 minutes to complete.

Researcher: ________________________ (name or initials of the interviewing investigator. Complete this box only if this questionnaire is carried out by telephone call or with the help of a researcher (interview). Otherwise, leave this box empty.)

Participant code: ________________________ (If you haven't receive your participant code, please contact the research staff at l.pantoja@upm.es, marcela.gonzalez.gross@upm.es, or gi.imfine@upm.es and request the unique participant code that you will use throughout the entire study as identification. If possible, memorize this code)

Today's date: ________________________ (DD/MM/YYYY format)

Date since you started following the recommended dietary instructions (provided during your visit): ________________________ (DD/MM/YYYY format)

1. **During the last month, have you followed all the dietary recommended indications?**
   - (1) Yes
   - (2) No (Select "No" if there are foodstuffs/allergens, outside the dietary recommended indications, that you have occasionally included in the diet)

2. **Specify below which of the restricted foodstuffs/allergens you have included in your diet during the last month:**

   Foodstuff/allergen 1: ________________________

   Choose the frequency of consumption of this foodstuff/allergen during the last month:

   - Never or rarely (none or 1 time during the last month)
   - Sometimes (2 or 3 times during the last month)
   - Frequently (4 or 5 times during the last month)
   - Very frequently (more than 5 times during the last month)
   - Always (daily or almost daily during the last month. I haven't avoided this foodstuff/allergen at all during the last month)

Foodstuff/allergen 2: ____________________

Choose the frequency of consumption of this foodstuff/allergen during the last month:

- Never or rarely (none or 1 time during the last month)
- Sometimes (2 or 3 times during the last month)
- Frequently (4 or 5 times during the last month)
- Very frequently (more than 5 times during the last month)
- Always (daily or almost daily during the last month. I haven't avoided this foodstuff/allergen at all during the last month)

Foodstuff/allergen 3: ____________________

Choose the frequency of consumption of this foodstuff/allergen during the last month:

- (1) Never or rarely (none or 1 time during the last month)
- (2) Sometimes (2 or 3 times during the last month)
- (3) Frequently (4 or 5 times during the last month)
- (4) Very frequently (more than 5 times during the last month)
- (5) Always (daily or almost daily during the last month. I haven't avoided this foodstuff/allergen at all during the last month)

(You can expand this list of foodstuffs/allergens as many times as necessary)

3. **After the last month, do you think you have improved in some symptomatology aspect?** (e.g., I am less constipated, I have noticed less redness on the skin, I am less depressed and anxious).
   - (1) Yes
   - (2) No

   If so (if selected "Yes"), specify in which specific symptomatology have you improved?

   ____

4. **During the last month, how many times have you been in the following situations?**

   4.a. Social events (parties), birthdays, weddings, and any other social event involving food and/or beverages):
   - (1) Never or rarely (none or 1 time during the last month)
   - (2) Sometimes (2 or 3 times during the last month)
   - (3) Frequently (4 or 5 times during the last month)
   - (4) Very frequently (more than 5 times during the last month)
   - (5) Always (daily or almost daily during the last month)

PARTICIPANT CODE: A L

RESEARCHERS: ____________________
(INITIALS OF THE RESEARCHERS)

4.b. Out-of-home eating:

- ○ (1) Never or rarely (none or 1 time during the last month)
- ○ (2) Sometimes (2 or 3 times during the last month)
- ○ (3) Frequently (4 or 5 times during the last month)
- ○ (4) Very frequently (more than 5 times during the last month)
- ○ (5) Always (daily or almost daily during the last month)

4.c. Presence and/or traces of one or more of your restricted foodstuffs/allergens:

- ○ (1) Never or rarely (none or 1 time during the last month)
- ○ (2) Sometimes (2 or 3 times during the last month)
- ○ (3) Frequently (4 or 5 times during the last month)
- ○ (4) Very frequently (more than 5 times during the last month)
- ○ (5) Always (daily or almost daily during the last month)

4.d. High fat diet

- ○ (1) Never or rarely (none or 1 time during the last month)
- ○ (2) Sometimes (2 or 3 times during the last month)
- ○ (3) Frequently (4 or 5 times during the last month)
- ○ (4) Very frequently (more than 5 times during the last month)
- ○ (5) Always (daily or almost daily during the last month)

4.e. High amount of food (e.g. free buffets, resort restaurants)

- ○ (1) Never or rarely (none or 1 time during the last month)
- ○ (2) Sometimes (2 or 3 times during the last month)
- ○ (3) Frequently (4 or 5 times during the last month)
- ○ (4) Very frequently (more than 5 times during the last month)
- ○ (5) Always (daily or almost daily during the last month)

5. Do you think it is worth maintaining this diet?

- ○ (1) Yes
- ○ (2) No

Why?

____

6. Do you think you feel able to maintain this diet?

- ○ (1) Yes
- ○ (2) No

Why?

____

7. Participant signature or initials:

____

PARTICIPANT CODE: A L

RESEARCHERS: ___________________

(INITIALS OF THE RESEARCHERS)

END OF THE DIETARY ADHERENCE QUESTIONNAIRE (DAQ)

THANK YOU VERY MUCH

REFERENCES:

1. Gila-Díaz A, Arribas SM, López de Pablo ÁL, López-Giménez MR, Phuthong S, Ramiro-Cortijo D. Development and Validation of a Questionnaire to Assess Adherence to the Healthy Food Pyramid in Spanish Adults. Nutrients. 2020; 12(6):1656.
2. Ruggeri S, Buonocore P, Amoriello T. New Validated Short Questionnaire for the Evaluation of the Adherence of Mediterranean Diet and Nutritional Sustainability in All Adult Population Groups. Nutrients. 2022 Dec 5;14(23):5177.

HOW TO CITE THIS DOCUMENT:

Pantoja-Arévalo L, Gesteiro E, Pérez-Ruiz M, *et al*. The multifactorial approach and the Food-Allergen Specific Substitutive Diet as a tool to manage and ameliorate adverse reactions to foodstuffs in adulthood: Study Protocol for a Randomized Controlled Trial. The ALASKA study. Supplementary Information. *Trials*. 2024.

PARTICIPANT CODE: A L _ _ _ _

RESEARCHERS: ______________________
(INITIALS OF THE RESEARCHERS)

ANNEX V. BLOOD SAMPLE QUESTIONNAIRE (BSQ)

INSTRUCTIONS:

- Complete the following Blood Sample Questionnaire (BSQ) right before the blood sample collection.
- Most of the questions refer to the participant status of the **last 24 hours** (unless otherwise specified).
- This questionnaire is divided in two sections: clinical and physical activity.
- The grey boxes are reserved to be fulfilled by the researcher (s). Do not complete the grey boxes.

Researcher: ______________________ (name or initials of the interviewing investigator. Complete this box only if this questionnaire is carried out with the help of a researcher (interview). Otherwise, leave this box empty.)

Participant code: ______________________ (If you haven't receive your participant code, please contact the research staff at l.pantoja@upm.es, marcela.gonzalez.gross@upm.es, or gi.imfine@upm.es and request the unique participant code that you will use throughout the entire study as identification. If possible, memorize this code)

Today's date: ______________________ (DD/MM/YYYY format)

Today's time: ______________________ (HH:MM format)

CLINICAL SECTION

1. **Sex (biological):**
   - (1) Man
   - (2) Woman

2. **Temperature (°C):** ______________________ (e.g., 36.3 Do not write units)

3. **Has the participant had fever of more than 38ºC during the last 24 hours?**
   - (1) Yes
   - (2) No

4. **Has the participant had any of the following signs and/or symptoms during the last 24 hours?**
   - (1) Cough
   - (2) Sneezing
   - (3) Nasal secretion
   - (4) Itchy skin
   - (5) Other similar sign and/or symptom
   - (6) None of the above signs and/or symptoms

   If "other similar sign and/or symptom" (option 5); which other similar sign or symptom have the participant had during the last 24 hours?

   ______________________ (e.g., nasal congestion)

POLITÉCNICA

PARTICIPANT CODE: A L

RESEARCHERS: ______________________

(INITIALS OF THE RESEARCHERS)

INEF

5. **Has the participant had any of the following pathologies and/or diseases during the last 24 hours?**
   - (1) Respiratory infections (flu, rhinitis, common cold, pharyngitis, laryngitis, pneumonia, tracheobronchitis)
   - (2) Herpes virus
   - (3) Asthma
   - (4) Urinary tract infection
   - (5) Other similar disease and/or pathology
   - (6) None of the above diseases and/or pathologies

   If "other similar disease and/or pathology" (option 5); which other similar sign or symptom have the participant had during the last 24 hours?

   ______________________ (e.g., influenza)

6. **Is the participant in a fasting condition of at least 8 hours?**
   - (1) Yes
   - (2) No

   If "no" (option 2), specify the current fasting hours of the participant:

   ______________________ (i.e., fasting hours of the participant, do not use units or words)

   If "no" (option 2), write below the date and time of the last food and/or beverage intake of the participant (except for water):

   ______________________ (DD/MM/YYYY HH:MM format)

7. **Has the participant had any complications (infection, hematoma, blood clots) in any blood extraction/ collection during the last year?**
   - (1) Yes
   - (2) No

   If "yes", briefly describe the complication or problem that the participant has had **in a blood extraction/ collection during the last year**:

   ______________________ (e.g. Infection in the extraction area blood)

   Note: Complete, as a researcher, the "Adverse Event" (Annex VII) in case of major complication during today's blood extraction/collection.

8. **Has the participant been vaccinated during the last 2 weeks?**
   - (1) Yes
   - (2) No

   If "yes", select the vaccine that the participant has received during the last 2 weeks:
   - (1) Flu
   - (2) Tetanus
   - (3) COVID-19
   - (4) Allergy
   - (5) Other vaccine

   Note: You can select more than 1 option for this question.

   If "other vaccine" (option 5), state the name of the vaccine that the participant has received during the past 2 weeks: ______________________

POLITÉCNICA

PARTICIPANT CODE: A L

RESEARCHERS: ____________________
(INITIALS OF THE RESEARCHERS)

INEF

9. Did the participant take any medication and/or food supplement during yesterday?

- (1) Yes
- (2) No

If "yes" (option 1), write below the medication and/or food supplement that the participant took yesterday and select below the frequency of consumption (you can also specify the concentration; e.g, paracetamol 1000mg 1/day):

A. ______________
- (1) 1/day
- (2) 2/day
- (3) 3/day
- (4) 4/day
- (5) > 5/day

B. ______________
- (1) 1/day
- (2) 2/day
- (3) 3/day
- (4) 4/day
- (5) > 5/day

C. ______________
- (1) 1/day
- (2) 2/day
- (3) 3/day
- (4) 4/day
- (5) > 5/day

D. ______________
- (1) 1/day
- (2) 2/day
- (3) 3/day
- (4) 4/day
- (5) > 5/day

If the participant is sex (2) woman; then answer questions from 10 to:

10. Select the current state of the participant:

- (1) Menstruation (menstrual cycle)
- (2) Pre-menopause (with medical diagnosis)
- (3) Menopause (with medical diagnosis)
- (4) Post-menopause (with medical diagnosis)

If "menstruation or menstrual cycle" (option 1), specify the date of the first day of the participant's menstruation: ____________________ (DD/MM/YYYY format)

If "pre-menopause (with medical diagnosis)" (option 2), specify the date of the first day of the participant's pre-menopause: ____________________ (DD/MM/YYYY format)

If "menopause (with medical diagnosis)" (option 3), specify the date of the first day of the participant's menopause: ______________________ (DD/MM/YYYY format)

If "post-menopause (with medical diagnosis)" (option 4), specify the date of the first day of the participant's post-menopause: ______________________ (DD/MM/YYYY format)

PARTICIPANT CODE: A L

RESEARCHERS: ____________________

(INITIALS OF THE RESEARCHERS)

PHYSICAL ACTIVITY

11. Select the type of transportation that the participant used today to arrive to the blood extraction appointment:

- (1) Car or taxi
- (2) Bus, subway or train
- (3) Motorcycle
- (4) Bicycle
- (5) Walking
- (6) Other type of transportation

Note: You can select more than 1 option for this question.

If "other type of transportation" (option 6), specify which other (s) type (s) of transportation did the participant used to arrive to the blood extraction appointment:

____________________ (e.g., electric scooter)

12. Did the participant perform any vigorous physical activity during the last 12 hours such as: lifting heavy objects, swimming, tennis, or any other team sport?

- (1) Yes
- (2) No

If "yes" (option 1), write below the type of vigorous physical activity:

____________________ (e.g., weightlifting, swimming, tennis)

Time of the vigorous physical activity (SINCE):

____________________ (HH:MM format)

Time of the vigorous physical activity (UNTIL):

____________________ (HH:MM format)

END OF THE BLOOD SAMPLE QUESTIONNAIRE (BSQ)

THANK YOU VERY MUCH

REFERENCES:

1. González-Gross, Marcela, et al. (ed.). Manual of operation The HELENA study. Universidad de Zaragoza, 2014, p262.

HOW TO CITE THIS DOCUMENT:

Pantoja-Arévalo L, Gesteiro E, Pérez-Ruiz M, *et al*. The multifactorial approach and the Food-Allergen Specific Substitutive Diet as a tool to manage and ameliorate adverse reactions to foodstuffs in adulthood: Study Protocol for a Randomized Controlled Trial. The ALASKA study. Supplementary Information. *Trials*. 2024.

ANNEX VI. BREATH TEST INSTRUCTIONS:

LACTOSE AND FRUCTOSE TEST

INSTRUCTIONS:

- Choose one morning for the lactose test and another morning, of a different day, for the fructose test.
- The lactose and fructose tests must be carried out in two different days.
- You will need 2 hours to take each test.
- This form has three sections: preparatory period, breath test kit content and test performance.
- If you have any doubt reagarding this instructions, please contact the research staff at l.pantoja@upm.es, marcela.gonzalez.gross@upm.es, or gi.imfine@upm.es

PREPARATORY PERIOD

1. Avoid taking antibiotics for a period of 7 days before performing the test.
2. Avoid taking probiotics (yogurt and fermented milk, kombucha, tempeh, sauerkraut, miso, pickles, kimchi, or similar) for a period of 4 to 5 days before the test.
3. Do not smoke at least 1 hour before starting the test or during the test.
4. Do not sleep or exercise at least 1 hour before the test or during the test.
5. Do not eat or drink anything other than filtered or bottled water during the test. You can drink as much water as you like before or during the test but do so in small sips of water rather than drinking large amounts at once.
6. Brush your teeth as usual before performing the test.
7. Avoid the following foods 24 hours before the test: cereals, bread, pastries, cookies, fruits (including natural juices), sugars and sweeteners (including natural and artificial), milk and dairy products, vegetables, nuts and seeds, chocolate, soy and derivatives and any alcoholic beverage.
8. The recommended dietary intake, 24 hours before performing the test, is: fish, chicken or turkey, pork, beef, lamb, sheep, rabbit, eggs; with boiled rice and plain coffee and tea.
   Note: You can season the food with just 1 pinch of salt and pepper and 1 tablespoon of extra virgin olive oil.
9. Fast 8 hours before the start of the test.

BREATH TEST KIT CONTENT

1. 20g of substrate bags (20g of fructose and 20g of lactose powder).
2. 10 vacuum-glass tubes (5 glass tubes for lactose and 5 glass tubes for fructose).
3. 10 stickers that will be placed after blowing into each of the tubes (stickers with space to fill in the blanks: participant code, substrate, number of the tube, date and time).
4. 2 padded transport envelopes (1 for each set of tubes: lactose and fructose).
5. 2 disposable devices for air collection.
6. 1 bottled water (500ml).
7. 1 cleaned and dried glass.
8. 1 cleaned and dried spoon.
9. 1 chronometer.

TEST PERFORMANCE

1. Make sure you meet the requirements of the "preparatory period" section and check that you have all the materials described in the "breath test kit content" section.
2. Fill a cleaned and dried glass with bottled water.
3. Pour all the content of 1 bag of substrate (lactose or fructose) into the glass and stir firmly using a cleaned and dried spoon.
   Note: Do not drink yet.
4. Blow into the air inlet hole, inflating the air bag completely of the disposable device for air collection. While the bag is inflated, insert the cannula from inside the cap into tube #1. Press the tube and blow at the same time for 5 seconds. Inhale air through your nose and exhale through your mouth into the hole of the device. Avoid inhaling the air in the collection bag with your mouth.
5. Fill and place the corresponding sticker on tube #1.
6. Immediately drink all the solution of step 3.
7. Start the timer for 30 minutes.
8. After 30 minutes, blow out, following the same instructions as steps 4 and 5 for tube #2. Fill and place the corresponding sticker on tube #2.
9. After 60 minutes blow out, following the same instructions as steps 4 and 5 for tube #3. Fill and place the corresponding sticker on tube #3.
10. After 90 minutes, blow out, following the same instructions as steps 4 and 5 for tube #4. Fill and place the corresponding sticker on tube #4.
11. After 120 minutes, blow out, following the same instructions as steps 4 and 5 for tube #5. Fill and place the corresponding sticker on tube #5.
12. You may take sips of bottled or filtered water whenever you like during the performance of all the test.
13. Finally, store the 5 blown tubes in the provided padded envelope.
14. Discard the device for air collection and the bag containing the used substrate.
15. Repeat the procedure on a different day for the remaining substrate.
16. Give the researcher during visit 2 the 2 unsealed padded envelopes, each with 5 blown tubes.

PARTICIPANT CODE: A L

RESEARCHERS: ___________________________
(INITIALS OF THE RESEARCHERS)

END OF THE BREATH TEST INSTRUCTIONS

THANK YOU VERY MUCH

REFERENCES:

1. QuinTron. QuinTron BreathTracker™ Digital Microlyzer SC and Lactose/Fructose Intolerance Hydrogen and Methane kit Instructions Manual: Quintron Instrument Company INC; 2020.

HOW TO CITE THIS DOCUMENT:

Pantoja-Arévalo L, Gesteiro E, Pérez-Ruiz M, *et al*. The multifactorial approach and the Food-Allergen Specific Substitutive Diet as a tool to manage and ameliorate adverse reactions to foodstuffs in adulthood: Study Protocol for a Randomized Controlled Trial. The ALASKA study. Supplementary Information. *Trials*. 2024.

PARTICIPANT CODE: A L

RESEARCHERS: ________________________
(INITIALS OF THE RESEARCHERS)

ANNEX VII. ADVERSE EVENT FORM

INSTRUCTIONS:

- Complete the following Adverse Event Form.
- The grey boxes are reserved to be fulfilled by the researcher (s). Do not complete the grey boxes.

Researcher: ________________________ (name or initials of the interviewing investigator. Complete this box only if this questionnaire is carried out with the help of a researcher (interview). Otherwise, leave this box empty.)

Participant code: ________________________ (If you haven't receive your participant code, please contact the research staff at l.pantoja@upm.es, marcela.gonzalez.gross@upm.es, or gi.imfine@upm.es and request the unique participant code that you will use throughout the entire study as identification. If possible, memorize this code)

Today's date: ________________________ (DD/MM/YYYY format)

Today's time: ________________________ (HH:MM format)

1. **How many adverse events have the participant experienced during this study?:**
   - (0) None
   - (1) 1
   - (2) 2
   - (3) More than 2

   If "1", "2", or "3" selected (options 1, 2 or 3), please specify:

   1.a. Date and time of the START of the adverse event 1:
   ________________________ (DD/MM/YYYY HH:MM format)

   1.b. Describe adverse event 1, indicating the consequences that the participant has suffered:
   ____

   1.c. Date and time of the END of the adverse event 1:
   ________________________ (DD/MM/YYYY HH:MM format)

   1.d. Date and time of the START of the adverse event 2:
   ________________________ (DD/MM/YYYY HH:MM format)

   1.e. Describe adverse event 2, indicating the consequences that the participant has suffered:
   ____

   1.f. Date and time of the END of the adverse event 2:
   ________________________ (DD/MM/YYYY HH:MM format)

PARTICIPANT CODE: A L

RESEARCHERS: ______________________

(INITIALS OF THE RESEARCHERS)

1.g. Date and time of the START of the adverse event 3:

______________________ (DD/MM/YYYY HH:MM format)

1.h. Describe adverse event 3, indicating the consequences that the participant has suffered:

____

1.i. Date and time of the END of the adverse event 3:

______________________ (DD/MM/YYYY HH:MM format)

SERIOUS ADVERSE EVENTS

2. Has the participant experienced a serious adverse event such as hospitalization, probability of death, or similar during this study?

- (1) Yes
- (2) No

If "yes" (option 1), specify:

2.a. Date and time of the START of the serious adverse event:

______________________ (DD/MM/YYYY HH:MM format)

2.b. Describe serious adverse event, indicating if it was hospitalization, probability of death, or similar and the signs and/or symptoms that the participant has suffered:

____

____

2.c. Date and time of the END of the serious adverse event:

______________________ (DD/MM/YYYY HH:MM format)

3. Researcher signature, name or initials:

______________________ (signature, name or initials of the researcher)

END OF THE ADVERSE EVENT FORM

THANK YOU VERY MUCH

REFERENCES:

1. Albers, Ulrike. Evolution and treatment of vitamin B12 deficiency as a risk factor for (cognitive and functional) neurodegenerative diseases in institutionalized elderly. Doctoral Thesis Tesis. 2012, p173.

HOW TO CITE THIS DOCUMENT:

Pantoja-Arévalo L, Gesteiro E, Pérez-Ruiz M, *et al*. The multifactorial approach and the Food-Allergen Specific Substitutive Diet as a tool to manage and ameliorate adverse reactions to foodstuffs in adulthood: Study Protocol for a Randomized Controlled Trial. The ALASKA study. Supplementary Information. *Trials*. 2024.

PARTICIPANT CODE: A L

RESEARCHERS: ______________________
(INITIALS OF THE RESEARCHERS)

ALASKA Study. ALLERGIES AND FOOD INTOLERANCES IN ADULTS AND ATHLETES

ANNEX VIII. END OF STUDY FORM

INSTRUCTIONS:

- Complete the following End of Study Form.
- The grey boxes are reserved to be fulfilled by the researcher (s). Do not complete the grey boxes.

Researcher: ______________________ (name or initials of the interviewing investigator. Complete this box only if this questionnaire is carried out with the help of a researcher (interview). Otherwise, leave this box empty.)

Participant code: ______________________ (If you haven't receive your participant code, please contact the research staff at l.pantoja@upm.es, marcela.gonzalez.gross@upm.es, or gi.imfine@upm.es and request the unique participant code that you will use throughout the entire study as identification. If possible, memorize this code)

Today's date: ______________________ (DD/MM/YYYY format)

1. **Termination date of participation in this study:**
   ______________________ (DD/MM/YYYY format)

2. **Does the participant end his/her participation in this study prematurely (before the study is officially completed?**
   - (1) Yes
   - (2) No

   If "yes" (option 1), select the reason(s) why the participant is leaving the study prematurely:

   - (1) Voluntary
   - (2) Non-compliance of the inclusion-exclusion criteria or decision of the researcher
   - (3) Adverse Event (complete the Adverse Event Form, Annex VII)
   - (4) Interrupted follow-up
   - (5) Other reason

   If "other reason" (option 5), write below which other reason does the participant has to leave the study prematurely:
   ______________________ (other reason)

3. **Respond about questionnaires and/or activities that have been completed, not completed, or partially completed by the participant:**

QUESTIONNAIRES

3.a. Informed consent
- (1) Completed
- (2) Non-completed
- (3) Partially completed

POLITÉCNICA

PARTICIPANT CODE: A L

RESEARCHERS: ____________________
(INITIALS OF THE RESEARCHERS)

3.b. Demographics and clinical information form
- (1) Completed
- (2) Non-completed
- (3) Partially completed

3.c. Inclusion/exclusion criteria form
- (1) Completed
- (2) Non-completed
- (3) Partially completed

3.d. Blood sample questionnaire (BSQ), M1 and M5/6.
- (1) Completed
- (2) Non-completed
- (3) Partially completed

3.e. Pathologies and Symptomatology Questionnaire associated with Adverse Reactions to Foodstuffs (PSIMP-RAA-10), M1 and M5/6.
- (1) Completed
- (2) Non-completed
- (3) Partially completed

3.f. Food and Beverages Frequency Consumption Questionnaire to Identify Adverse Reactions to Foodstuffs (FBFC-ARFSQ-18), M1 and M5/6.
- (1) Completed
- (2) Non-completed
- (3) Partially completed

3.g. International Physical Activity Questionnaire Long Form (IPAQ-LF), M1 and M5/6.
- (1) Completed
- (2) Non-completed
- (3) Partially completed

3.h. Short-Form Health Survey 12 (SF-12), M1 and M5/6.
- (1) Completed
- (2) Non-completed
- (3) Partially completed

3.i. Food Allergy Quality of Life Questionnaire - Adult Form (FAQLQ-AF), M1 and M5/6.
- (1) Completed
- (2) Non-completed
- (3) Partially completed

3.j. Adverse event form.
- (1) Completed
- (2) Non-completed
- (3) Partially completed

PARTICIPANT CODE: A L _ _ _ _

RESEARCHERS: ______________

(INITIALS OF THE RESEARCHERS)

3.k. End of study form.
- (1) Completed
- (2) Non-completed
- (3) Partially completed

ACTIVITIES

3.l. Blood extraction/collection, M1 and M5/6.
- (1) Completed
- (2) Non-completed
- (3) Partially completed

3.m. Body composition, M1 and M5/6.
- (1) Completed
- (2) Non-completed
- (3) Partially completed

3.n. Height, M1 and M5/6.
- (1) Completed
- (2) Non-completed
- (3) Partially completed

3.o. Blood pressure, M1 and M5/6.
- (1) Completed
- (2) Non-completed
- (3) Partially completed

3.p. Åstrand-Ryhming Step Test (stÅ-R), M1 and M5/6.
- (1) Completed
- (2) Non-completed
- (3) Partially completed

3.q. Waist-rip ratio, M1 and M5/6.
- (1) Completed
- (2) Non-completed
- (3) Partially completed

3.r. Dinamometry, M1 and M5/6.
- (1) Completed
- (2) Non-completed
- (3) Partially completed

3.s. Sit-to-stand test, M1 and M5/6.
- (1) Completed
- (2) Non-completed
- (3) Partially completed

POLITÉCNICA

PARTICIPANT CODE: A L

RESEARCHERS: ______________________
(INITIALS OF THE RESEARCHERS)

INEF

3.t. Lactose breath test, M1 and M5/6.
- (1) Completed
- (2) Non-completed
- (3) Partially completed

3.u. Fructose breath test, M1 and M5/6.
- (1) Completed
- (2) Non-completed
- (3) Partially completed

3.v. 6-month Dietary intervention
- (1) Completed
- (2) Non-completed
- (3) Partially completed

Here you can describe the details of the uncompleted and partially completed activities by the participant. Use one line for each activity.

____
____
____
____
____
____
____
____

Reminder/rectifications

Regarding your data, the instructions provided by you in the initial Informed Consent will be followed. However, you can also rectify any related details, in this space.

____
____

4. Researcher signature, name or initials:

______________________ (signature, name or initials of the researcher)

PARTICIPANT CODE: A L

RESEARCHERS: ________________________
(INITIALS OF THE RESEARCHERS)

END OF THE END OF STUDY FORM

THANK YOU VERY MUCH

REFERENCES:

1. Albers, Ulrike. Evolution and treatment of vitamin B12 deficiency as a risk factor for (cognitive and functional) neurodegenerative diseases in institutionalized elderly. Doctoral Thesis Tesis. 2012, p174.

HOW TO CITE THIS DOCUMENT:

Pantoja-Arévalo L, Gesteiro E, Pérez-Ruiz M, *et al*. The multifactorial approach and the Food-Allergen Specific Substitutive Diet as a tool to manage and ameliorate adverse reactions to foodstuffs in adulthood: Study Protocol for a Randomized Controlled Trial. The ALASKA study. Supplementary Information. *Trials*. 2024.
