# Supplementary material for: The multifactorial approach and the food allergen-specific substitutive diet as a tool to manage and ameliorate adverse reactions to foodstuffs in adulthood: study protocol for a randomized controlled trial—the ALASKA study
Source: Trials. 2024 Jul 20;25:494. doi: 10.1186/s13063-024-08307-2 (PMC11264907; doi:10.1186/s13063-024-08307-2)
Supplement: Supplementary file 2 — Additional file 2. SPIRIT checklist. [file 13063_2024_8307_MOESM2_ESM.pdf]

SPIRIT Checklist for *Trials*

Complete this checklist by entering the page and line numbers where each of the items listed below can be found in your manuscript.

Your manuscript may not currently address all the items on the checklist. Please modify your text to include the missing information. If you are certain that an item does not apply, please state "n/a" and provide a short explanation. **Leaving an item blank or stating "n/a" without an explanation will lead to your manuscript being returned before review.**

Upload your completed checklist as an additional file when you submit to *Trials*. You must reference this additional file in the main text of your protocol submission. The completed SPIRIT figure must be included within the main body of the protocol text and can be downloaded here: http://www.spirit-statement.org/schedule-of-enrolment-interventions-and-assessments/

In your methods section, please state that you used the SPIRIT reporting guidelines, and cite them as:

Chan A-W, Tetzlaff JM, Gøtzsche PC, Altman DG, Mann H, Berlin J, Dickersin K, Hróbjartsson A, Schulz KF, Parulekar WR, Krleža-Jerić K, Laupacis A, Moher D. SPIRIT 2013 Explanation and Elaboration: Guidance for protocols of clinical trials. BMJ. 2013;346:e7586

		Reporting Item	Page and Line Number	Reason if not applicable
**Administrative information**				
Title	#1	Descriptive title identifying the study design, population, interventions, and, if applicable, trial acronym	Page 2, lines 1-3	**The multifactorial approach and the Food-Allergen Specific Substitutive Diet as a tool to manage and ameliorate adverse reactions to foodstuffs in adulthood: Study Protocol for a Randomized Controlled Trial. The ALASKA study.**
Trial registration	#2a	Trial identifier and registry name. If not yet registered, name of intended registry	Page 16, lines 335-338	**The protocol has been registered on ClinicalTrials.gov (Clinical Trials ID NCT05802017, name: "Relation**

				**Between Adverse Reactions to Food, Physical Performance and Health in a Mediterranean Population”).**
Trial registration: data set	#2b	All items from the World Health Organization Trial Registration Data Set	n/a	The trial have not been registered in the WHO; however, our registration accomplishes all the minimum standard list of items to be included in a trial registry. Our study have been registered in ClinicalTrials.gov (Clinical Trials ID NCT05802017, name: “Relation Between Adverse Reactions to Food, Physical Performance and Health in a Mediterranean Population”). (Page 16, lines 334-337)
Protocol version	#3	Date and version identifier	Page 16, line 336	Our protocol has only one final version. The protocol was constructed carefully resulting in 26 draft versions before sending the final version to the Ethics Committee of the Universidad Politécnica de Madrid. **The protocol has been approved by the Ethics Committee of the UPM (reference number 20200602, date: 17/07/2020).**
Funding	#4	Sources and types of financial, material, and other support	Page 34, lines 677-691	**AESKUBLOTS® kits will be manufactured by Aesku.Diagnostics GmbH (Aesku.Diagnostics GmbH, Wendelsheim, Germany). The design, management, analysis and reporting of the study are entirely independent of**

				**the manufacturers of AESKUBLOTS® kits (Aesku.Diagnostics GmbH, Wendelsheim, Germany).**
Roles and responsibilities: contributorship	#5a	Names, affiliations, and roles of protocol contributors	Page 2, lines 4-13	
Roles and responsibilities: sponsor contact information	#5b	Name and contact information for the trial sponsor	Page 34, line 688-691<br><br>(See Funding section)	**The ALASKA study RCT is conducted in the ImFINE Research Group (gi.imfine@upm.es) of the Universidad Politécnica de Madrid.**
Roles and responsibilities: sponsor and funder	#5c	Role of study sponsor and funders, if any, in study design; collection, management, analysis, and interpretation of data; writing of the report; and the decision to submit the report for publication, including whether they will have ultimate authority over any of these activities	Page 34, lines 677-691<br><br>(See Funding section)	
Roles and responsibilities: committees	#5d	Composition, roles, and responsibilities of the coordinating centre, steering committee, endpoint adjudication committee, data management team, and other individuals or groups overseeing the trial, if applicable (see Item 21a for data monitoring committee)	Pages 35-36, lines 723-786	
**Introduction**				
Background and rationale	#6a	Description of research question and justification for undertaking the trial, including summary of relevant studies (published and	Page 7-8, lines 104-153<br><br>(See Introduction section)	

		unpublished) examining benefits and harms for each intervention		
Background and rationale: choice of comparators	#6b	Explanation for choice of comparators	Page 8, lines 136-148	**The application of treatments such as strict food-allergen avoidance may cause nutritional deficiencies in certain cases of multiple food-allergen response due to the avoidance of important food groups. For instance, milk avoidance in adults may result in calcium deficiency (13) and lower intakes of zinc and vitamin B2. Likewise, prolonged gluten-free diet make individuals prone to the development of, most notably folate deficiency and lower intakes of calcium, iron, zinc, folate and fiber; affecting their nutritional status and altering the intestinal microbiota (14). Substitutive diets suggesting the replacement of allergy-related food for allergen-free food have shown symptoms' score improvement. Hence, the six-month intervention of the ALASKA study, to manage and ameliorate ARFS in adulthood, consists in the implementation of a general nutritional advice with dietary recommendations for a healthy lifestyle for subjects in the control group; and a six-month food-allergen specific substitutive diet**

				**(FASSD) for subjects in the intervention group. These effects are greater in subjects with specific pathologies: atopic dermatitis (15), urticaria (16) and gastrointestinal disorders (17).**
Objectives	#7	Specific objectives or hypotheses	Page 8, lines 149-153	The aim of this study is a) to develop a multifactorial strategy for ARFS management in adults with FA and/or FI; b) to describe the multiple influential variables in ARFS within the realm of ARFS management; and c) to design a personalized FASSD, as a six-month dietary treatment option for adults with ARFS and as a component of ARFS management.
Trial design	#8	Description of trial design including type of trial (eg, parallel group, crossover, factorial, single group), allocation ratio, and framework (eg, superiority, equivalence, non-inferiority, exploratory)	Page 10, lines 174-179 (Figure 2)<br><br>(See Study design section)	
**Methods: Participants, interventions, and outcomes**				
Study setting	#9	Description of study settings (eg, community clinic, academic hospital) and list of countries where data will be collected. Reference to where list of study sites can be obtained	Page 10, line 177	**This will be a randomized controlled trial (RCT), the Allergies and Food Intolerances in Adults and Athletes study (ALASKA study), conducted in the Universidad Politécnica de Madrid (UPM), located in the Region of Madrid, Spain.**

Eligibility criteria	#10	Inclusion and exclusion criteria for participants. If applicable, eligibility criteria for study centres and individuals who will perform the interventions (eg, surgeons, psychotherapists)	Pages 13-14, lines 252-278<br><br>(See Inclusion criteria and Exclusion criteria sections)<br><br>Page 16, lines 342-345	**The ALASKA study will be carried out by qualified professionals for medical, clinical, nutrition and physical activity and sport sciences practice of the ImFINE Research Group of the Faculty of Physical Activity and Sport Sciences of the UPM.**
Interventions: description	#11a	Interventions for each group with sufficient detail to allow replication, including how and when they will be administered	Pages 14-15 lines 296-319	
Interventions: modifications	#11b	Criteria for discontinuing or modifying allocated interventions for a given trial participant (eg, drug dose change in response to harms, participant request, or improving / worsening disease)	Page 12, lines 222-227	**Due to the nature of the dietary interventions, modifying allocated interventions is not foreseen in this study. However, if a subject chooses to discontinue the intervention on their own or if the researcher decides to stop a participant's intervention for any reason, it will be documented in each participant's individual case report and in the End of Study Form (Annex VIII). Subjects will be able to explain, or not, the reason for voluntarily abandoning the study.**
Interventions: adherance	#11c	Strategies to improve adherence to intervention protocols, and any procedures for monitoring adherence (eg, drug tablet return; laboratory tests)	Page 15, lines 311-315<br><br>Page 23-24, lines 397-400	**Additionally, to encourage subjects to follow the assigned six-month intervention and promote the dietary treatment adherence, all subjects will be contacted monthly during the study**

				**using a Dietary Adherence Questionnaire (Annex IV, authors' own creation, based on questions of previously published validated questionnaires (31) and adapted to the six-month intervention).**
Interventions: concomitant care	#11d	Relevant concomitant care and interventions that are permitted or prohibited during the trial	Page 15, lines 316-319<br><br>(See Six-month intervention section)<br><br>(Appendix 2. Inclusion and exclusion criteria form)	
Outcomes	#12	Primary, secondary, and other outcomes, including the specific measurement variable (eg, systolic blood pressure), analysis metric (eg, change from baseline, final value, time to event), method of aggregation (eg, median, proportion), and time point for each outcome. Explanation of the clinical relevance of chosen efficacy and harm outcomes is strongly recommended	Page 22, lines 367-372 (Figure 3)<br><br>Page 22-30, lines 367-575<br><br>(See Main variables section)	
Participant timeline	#13	Time schedule of enrolment, interventions (including any run-ins and washouts), assessments, and visits for participants. A schematic diagram is highly recommended (see Figure)	Page 10, lines 149-154 (Figure 2) (See Study design section)<br><br>+<br><br>Pages 16-19, line 280 (Table 1)	
Sample size	#14	Estimated number of participants needed to achieve study objectives and how it was	**Page 12, lines 211-216**	

		determined, including clinical and statistical assumptions supporting any sample size calculations	**Loss to follow-up:**<br>**Page 12, lines 217-227**<br>**(See section Sample size and missing data)**	
Recruitment	#15	Strategies for achieving adequate participant enrolment to reach target sample size	Page 11, lines 181-209<br>(See Recruitment section)	
**Methods: Assignment of interventions (for controlled trials)**				
Allocation: sequence generation	#16a	Method of generating the allocation sequence (eg, computer-generated random numbers), and list of any factors for stratification. To reduce predictability of a random sequence, details of any planned restriction (eg, blocking) should be provided in a separate document that is unavailable to those who enrol participants or assign interventions	Page 14, lines 279-295<br>(See Randomization and Allocation sections)	**The ALASKA study will follow a sample randomization using the RedCap® randomization automated tool (RRAT) for the study allocation of subjects.**
Allocation concealment mechanism	#16b	Mechanism of implementing the allocation sequence (eg, central telephone; sequentially numbered, opaque, sealed envelopes), describing any steps to conceal the sequence until interventions are assigned	Page 14, lines 281-283	**RRAT assigns subjects by chance (rather than by choice) into specific groups (Group A and Group B) and monitors the overall allocation progress and assignment of randomized subjects.**
Allocation: implementation	#16c	Who will generate the allocation sequence, who will enrol participants, and who will assign participants to interventions	Page 14, lines 289-291	**Subjects will be randomly placed in Group A (control), or Group B (intervention) in a 1.5 allocation ratio using the RRAT tool. RRAT tool access**

				**will be granted to the outcome researchers of the Alaska study.**
Blinding (masking)	#17a	Who will be blinded after assignment to interventions (eg, trial participants, care providers, outcome assessors, data analysts), and how	Page 14, lines 283-287	**It is not possible to blind subjects or staff in this study from the tested treatment due to nutritional interventions which will be used as dietary treatments for the management of ARFS. Nevertheless, the outcome assessors will be blinded to the group assignment given the use of anonymized participant codes among all the steps of the study.**
Blinding (masking): emergency unblinding	#17b	If blinded, circumstances under which unblinding is permissible, and procedure for revealing a participant's allocated intervention during the trial	n/a	**Unblinding of the outcome assessors is not foreseen in this study.**
**Methods: Data collection, management, and analysis**				
Data collection plan	#18a	Plans for assessment and collection of outcome, baseline, and other trial data, including any related processes to promote data quality (eg, duplicate measurements, training of assessors) and a description of study instruments (eg, questionnaires, laboratory tests) along with their reliability and validity, if known. Reference to where data collection forms can be found, if not in the protocol	Pages 18-21, line 366 (Table 2)<br>Pages 34-35, lines 705-722 (See Supplementary information section)	

Data collection plan: retention	#18b	Plans to promote participant retention and complete follow-up, including list of any outcome data to be collected for participants who discontinue or deviate from intervention protocols	Page 15, lines 311-315<br><br>Page 23-24, lines 397-400<br><br>(Same as 11c)<br><br>Page 29, lines 547-552<br><br>(See End of the study and adverse events Section)	**Additionally, to encourage subjects to follow the assigned six-month intervention and promote the dietary treatment adherence, all subjects will be contacted monthly during the study using a Dietary Adherence Questionnaire (Annex IV, authors' own creation, based on questions of previously published validated questionnaires (31) and adapted to the six-month intervention).**
Data management	#19	Plans for data entry, coding, security, and storage, including any related processes to promote data quality (eg, double data entry; range checks for data values). Reference to where details of data management procedures can be found, if not in the protocol	Pages 12-13, lines 228-251<br><br>(See Data storage Section)	
Statistics: outcomes	#20a	Statistical methods for analysing primary and secondary outcomes. Reference to where other details of the statistical analysis plan can be found, if not in the protocol	Pages 30-31, lines 576-600<br><br>(See Statistical analysis Section)	
Statistics: additional analyses	#20b	Methods for any additional analyses (eg, subgroup and adjusted analyses)	Page 31, lines 590-596<br><br>(See Statistical analysis Section)	
Statistics: analysis population and missing data	#20c	Definition of analysis population relating to protocol non-adherence (eg, as randomised	Page 31, lines 597-600	**Valid data values will be considered of subjects of study accomplishing at least main variables from 1 to 4 and from 6**

		analysis), and any statistical methods to handle missing data (eg, multiple imputation)		**to 7 (See Main Variables Section), the rest of the data values will be carefully either cleaned or imputed as missing values. Data will be analyzed using IBM-SPSS® Statistics software, version 26.0, and statistical significance will be set at 0.05.**
**Methods: Monitoring**				
Data monitoring: formal committee	#21a	Composition of data monitoring committee (DMC); summary of its role and reporting structure; statement of whether it is independent from the sponsor and competing interests; and reference to where further details about its charter can be found, if not in the protocol. Alternatively, an explanation of why a DMC is not needed	See Ethical Approval Document 2: Data management/monitoring approval.<br><br>The management of the data was approved by the Ethics Committee of the Universidad Politécnica de Madrid. Additionally, RedCap tool (https://redcap.cesvima.upm.es) is a clinical data management tool approved by the Ethics Committee of the Universidad Politécnica de Madrid for this clinical trial.	
Data monitoring: interim analysis	#21b	Description of any interim analyses and stopping guidelines, including who will have access to these interim results and make the final decision to terminate the trial	Pages 12-13, lines 238-242	**RedCap platform provides the "Data Exports, Reports, and Stats" module, which allows to view reports, inspect plots and descriptive statistics of the data, at any point/time of the trial, as**

				**well as export data to Microsoft Excel, SAS, Stata, R, or SPSS for analysis (if access provided by M.G.-G to the researchers of the Alaska study).**
Harms	#22	Plans for collecting, assessing, reporting, and managing solicited and spontaneously reported adverse events and other unintended effects of trial interventions or trial conduct	Page 29, lines 548-552	**9) End of the study and adverse events**<br><br>**Any potential negative outcomes linked to the dietary changes (minimal to zero risk probability) of the ALASKA study intervention, if any, will be documented using Annexes VII and VIII. The UPM will assist with an available medical physician, part of the ALASKA study researchers (M.P.-R.), to cover for any emergency associated with any step of this study protocol and will be present during all the clinical and physical assessments.**
Auditing	#23	Frequency and procedures for auditing trial conduct, if any, and whether the process will be independent from investigators and the sponsor	Page 13, lines 243-246	**Routine day-to-day audits; weekly back-ups of the datasets; and monthly internal audits of the available services (water and electricity) and resources (laboratory equipment) will be performed to support data quality.**
**Ethics and dissemination**				
Research ethics approval	#24	Plans for seeking research ethics committee / institutional review board (REC / IRB) approval	Page 16, 332-358	

			(See Ethical considerations and clinical trial registration section)	
Protocol amendments	#25	Plans for communicating important protocol modifications (eg, changes to eligibility criteria, outcomes, analyses) to relevant parties (eg, investigators, REC / IRBs, trial participants, trial registries, journals, regulators)	Page 17, Lines 351-358	
Consent or assent	#26a	Who will obtain informed consent or assent from potential trial participants or authorised surrogates, and how (see Item 32)	Page 16, lines 338-340<br><br>**Appendix 1. Informed consent model for the ALASKA study**	**A signed informed consent model will be obtained from all study participants before their participation commencement**.
Consent or assent: ancillary studies	#26b	Additional consent provisions for collection and use of participant data and biological specimens in ancillary studies, if applicable	Page 16, lines 345-347<br><br>(Appendix 1. Informed consent form)	**…Besides, participants will be asked whether they authorize the researchers of this study to use their samples for subsequent genetic tests and/or future studies (Appendix 1. Informed consent model for the ALASKA study)**
Confidentiality	#27	How personal information about potential and enrolled participants will be collected, shared, and maintained in order to protect confidentiality before, during, and after the trial	Pages 13, lines 242-243	**Complete individual records will be documented using the unique and anonymous participant code and will not contain identifiable participants' information.**
Declaration of interests	#28	Financial and other competing interests for principal investigators for the overall trial and each study site	Page 34, 692-695	**Declaration of Interests**<br><br>**PW and TM are part of Aesku.Diagnostics GmbH staff.**

				**Aesku.Diagnostics GmbH did not participate in the design of the protocol, nor in the analysis and interpretation of the data outcomes. The rest of the authors have nothing to report.**
Data access	#29	Statement of who will have access to the final trial dataset, and disclosure of contractual agreements that limit such access for investigators	Page 12, 234-238	**Databases will be locked using a secure password and with access to the coordinator and principal investigator (PI) of the ALASKA study (L.P.-A., M.G.-G). Additional researchers, after the authorization of the PI (M.G.-G), will have also access to the password and the study databases. M.G.-G will make final decisions regarding the RedCap® platform management, trial supervision and the trial start/termination point.**
Ancillary and post trial care	#30	Provisions, if any, for ancillary and post-trial care, and for compensation to those who suffer harm from trial participation	Page 29, lines 547-552<br>(See End of the study and adverse events section)<br>Page 29-30, lines 553-569<br>(See (Optional) Repetition of the assessments 2 to 8 and intervention adjustment Section)	
Dissemination policy: trial results	#31a	Plans for investigators and sponsor to communicate trial results to participants,	Page 33, lines 672-676	**No later than 3 years after the collection of 1-year post-randomization**

		healthcare professionals, the public, and other relevant groups (eg, via publication, reporting in results databases, or other data sharing arrangements), including any publication restrictions		**processes, publications (articles, abstracts and other manuscripts) explaining and evaluating the study outcomes will be delivered.**
Dissemination policy: authorship	#31b	Authorship eligibility guidelines and any intended use of professional writers	Page 34, lines 696-704<br><br>(See Authors' contributions section)	
Dissemination policy: reproducible research	#31c	Plans, if any, for granting public access to the full protocol, participant-level dataset, and statistical code	Page 33, lines 672-676<br><br>(See Data sharing statement section)	**Data sharing statement**<br><br>**…. Public access to the present protocol publication and its subsequent publications will be available at the local projects section of the ImFINE Research Group website (https://short.upm.es/kwgpo).**
**Appendices**				
Informed consent materials	#32	Model consent form and other related documentation given to participants and authorised surrogates	**n/a**<br><br>**See Appendix 1. Informed consent model for the ALASKA study**	**Not applicable - no identifying images or other personal or clinical details of participants are presented here or will be presented in reports of the trial results. The participant information materials and informed consent form are attached**
Biological specimens	#33	Plans for collection, laboratory evaluation, and storage of biological specimens for genetic or	Page 25, lines 434-438	**Remaining samples from subjects who offer their consent (serum, plasma and whole blood) will be stored in 1ml**

		molecular analysis in the current trial and for future use in ancillary studies, if applicable		**aliquots at −80°C (ultrafreezing conditions). Colored 2ml microtubes will be used for the ultrafreezing storage of 1ml aliquots of serum (yellow, REF05-408-140), plasma (green, REF05-408-142) and whole blood (red, REF05-408-139) (Thermo Fisher Scientific Inc., Waltham, Massachusetts, USA).**

It is strongly recommended that this checklist be read in conjunction with the SPIRIT 2013 Explanation & Elaboration for important clarification on the items. Amendments to the protocol should be tracked and dated. The SPIRIT checklist is copyrighted by the SPIRIT Group under the Creative Commons "Attribution-NonCommercial-NoDerivs 3.0 Unported" license. This checklist can be completed online using https://www.goodreports.org/, a tool made by the EQUATOR Network in collaboration with Penelope.ai
