# Supplementary material for: The multifactorial approach and the food allergen-specific substitutive diet as a tool to manage and ameliorate adverse reactions to foodstuffs in adulthood: study protocol for a randomized controlled trial—the ALASKA study
Source: Trials. 2024 Jul 20;25:494. doi: 10.1186/s13063-024-08307-2 (PMC11264907; doi:10.1186/s13063-024-08307-2)
Supplement: Supplementary file 3 — Additional file 3: Appendix 1. Informed consent model for the ALASKA study. [file 13063_2024_8307_MOESM3_ESM.pdf]

POLITÉCNICA

PARTICIPANT CODE: A L

RESEARCHERS: ________________________

(INITIALS OF THE RESEARCHERS)

INEF

APPENDIX 1. INFORMED CONSENT MODEL

INSTRUCTIONS:

Welcome to the ALASKA study on Food Allergies and Intolerances in sedentary and athletic adults.
Please read carefully and sign the following Informed Consent.

Participant code: ________________________ (If you haven't receive your participant code, please contact the research staff at l.pantoja@upm.es, marcela.gonzalez.gross@upm.es, or gi.imfine@upm.es and request the unique participant code that you will use throughout the entire study as identification. If possible, memorize this code)

Today's date: ________________________ (DD/MM/YYYY format)

Sex (biological):

- ○ (1) Man
- ○ (2) Woman

Age: ________________________

Date of birth: ________________________ (DD/MM/YYYY format)

INFORMED CONSENT

Dear participant:

The ImFINE Research Group, of the Universidad Politécnica de Madrid (UPM) is carrying out a research project that aims to determine the impact of the adverse reactions to foodstuffs (ARFS), such as food allergies and food intolerances, over the quality of life of adults of the Mediterranean population with different levels of physical activity.

The study is carried out following current legislation on research activities in humans, the guidelines of the Declaration of Helsinki on ethical principles in research and has been approved by the Committee of Ethics of the Polytechnic University of Madrid (UPM) dated 07-20-2020 and reference number 20200602. The personal data of all participants will be treated in accordance with Regulation (EU) 2016/679, of 27 April 2016 and Royal Decree 1720/2007, of December 21, which approves the Development Regulation of Organic Law 15/1999, of December 13, Protection of Personal Data and Law 14/2007 of Biomedical Research.

You have been invited to participate in this study. Please find below the information regarding your decision to voluntarily participate. Use as much time as you want to study the content of this document before deciding whether to participate in this study or not.

ALASKA STUDY INFORMATION

The ALASKA study lasts approximately 5 to 6 months, and your participation will consist of:

- Responding to validated questionnaires related to the objective of the study.
- Clinical measurements, including: venous blood analysis and breath test. Physical tests, including: measurement of body composition, body perimeters, blood pressure, heart rate and a battery of physical exercises.
- Accomplishment of dietary recommendations as requested by the researchers of this study.

PARTICIPANT CODE: A L

RESEARCHERS: ______________________
(*INITIALS OF THE RESEARCHERS*)

CLINICAL MEASUREMENTS

- Blood test: venous blood extraction under fasting conditions to carry out hematological, biochemical and immunological analysis (and subsequent genetic tests if authorized by the participant).
- Breath test: Analysis of the concentration of exhaled gases of Hydrogen (H_2) and Methane (CH_4) through the collection of expired gas samples (blow into a disposable air collection device) for subsequent determination of food intolerance to lactose and/or fructose.

PHYSICAL TESTS

- Blood pressure and heart rate: measurement of the blood circulation intensity in the bloodstream and the number of times the heart beats per minute using a medical blood pressure monitor.
- Electrical bioimpedance: body composition analysis using an electrical bioimpedance scale. Note: If you have a pacemaker, metal prosthesis or are pregnant, please notify the researcher through gi.imfine@upm.es . Or instead, answer carefully the questions in the next section.
- Muscular function: determination of muscle strength, balance and speed of getting up of a chair (assessment of equilibrium) using a calibrated platform.
- Dynamometry: monitoring of physical condition using a hand-dynamometer that measures hand grip strength, assessing the contraction of the intrinsic and extrinsic muscles of the hand.
- Aerobic capacity: estimation of maximum oxygen consumption, VO_2 max, using heart rate measurement after performing aerobic exercise for a period of 5 minutes (Åstrand-Ryhming Step Test, stÅ-R).

RISKS

- The collection of blood and breath samples will be carried out by a qualified professional for clinical practice, so it signifies minimal to no risk for the participant.
- Your diet and exercise habits could moderately change. However, these changes will not represent dietary or physical alterations that could threaten against your health and will be monitored by a qualified professional for nutritional practice.
- In case of participants presenting metals allergies with potential adverse effects such as atopic dermatitis due to contact with metals (nickel, cobalt, chromium, among others), please notify the researcher through gi.imfine@upm.es. Or instead, answer carefully the questions in the next section.

GUARANTEE

- Active communication by the researcher with the participant.
- The physical tests will be carried out by a qualified professional in physical activity and sports sciences practice.
- Pseudonymization and safeguarding of data on a secure platform called REDCap. REDCap is a secure web platform and electronic data capture tool hosted at the Supercomputing and Visualization Center of Madrid of the Universidad Politécnica de Madrid (CESVIMA-UPM).
- Clinical samples obtained from the participant will be stored under frozen conditions (-20ºC) to subsequent genetic tests and ultra-freezing (-80ºC) for storage in the Faculty of Physical Activity and Sports Sciences (INEF-UPM) and the health center of the Spanish Health Protection Agency in Sports (AEPSAD) for academic and research purposes.
- The processed results will be published in scientific journals always in an anonymized format. The pseudonymized data files will be destroyed after being kept for a period of 10 years.
- At the end of the study we will confidentially provide you with a digital report containing the personal results obtained in the clinical and physical tests that you have done.

PARTICIPANT CODE: A L _ _ _ _

RESEARCHERS: ______________________

(INITIALS OF THE RESEARCHERS)

Answer the following questions according to your current status and physiological condition:

Are you currently pregnant?:

- (1) Yes
- (2) No

Number of weeks of pregnancy: ______________________ (current week of pregnancy)

Do you have a pacemaker, prosthesis or metal implants?:

- (1) I have a pacemaker
- (2) I have metal prostheses or implants
- (3) I have a pacemaker and prosthetics or metal implants
- (4) I wear something similar
- (5) I do not have a pacemaker or prosthesis or metal implants or anything similar.

You have selected "I wear something similar", write:
What similar device do you wear?: ______________________

Do you have a metal allergy (nickel, cobalt, chromium, among others)?:

- (1) Yes
- (2) No

You have selected that you have a metal allergy, write:
The type of metal you are allergic to: ______________________ (e.g. nickel, cobalt, chromium)

Are you advised against physical exercise by your doctor?:

- (1) Yes
- (2) No

Do you have any limitations that prevent you from performing physical exercise safely?:

- (1) Yes
- (2) No

Have you ever suffered cardiac arrest, angina pectoris or heart failure?:

- (1) Yes
- (2) No

When you exercise, have you had chest pain, chest tightness, or dizziness?:

- (1) Yes
- (2) No

SUMMARY OF THE ALASKA STUDY CHARACTERISTICS

Your data will be anonymously used for research purposes only. Even if you agree to voluntarily participate in this study, you can abandon this study at any time, without having to justify yourself, and of course, without any fear of being penalized. Likewise, the researcher reserves the right to terminate your participation if the

PARTICIPANT CODE: A L

RESEARCHERS: ______________________
(INITIALS OF THE RESEARCHERS)

researcher consider that it is for the benefit of the study and/or the participant. You will not get any financial benefits for participating in this study, although you will receive benefits in terms of dietary healthy advice, exercise and lifestyle based on your metabolic status. The participation in this study requires no cost for you.

Once you have carefully read the above information, please sign the following document:

INFORMED CONSENT ACT

I ______________________ (name) ______________________ (lastname), agree to voluntarily and anonymously participate in the Research Project study entitled: RELATIONSHIP BETWEEN ADVERSE REACTIONS TO FOOD, PHYSICAL PERFORMANCE AND HEALTH IN MEDITERRANEAN POPULATION (ALASKA study), developed by BSc. Eng. MSc. LISSET PANTOJA-ARÉVALO, predoctoral researcher of the Doctorate program in PHYSICAL ACTIVITY AND SPORTS SCIENCES at the Universidad Politécnica de Madrid, supervised by Prof. Dr. MARCELA GONZÁLEZ-GROSS, Director of the ImFine Research Group (Improvement of Health by Fitness, Nutrition and Exercise Research Group) of the Faculty of Physical Activity and Sports Sciences-INEF of the Universidad Politécnica de Madrid and co-directed by Dr. PharmD. EVA GESTEIRO ALEJOS, Permanent Professor of the Faculty of Physical Activity and Sports Sciences-INEF of the Universidad Politécnica de Madrid.

____

(Identity Document Number (without letter))

I declare that:

- I consent to perform all physical and clinical tests, including those requiring venous blood extraction and the collection of breath samples and answer all necessary questionnaires to carry out the present investigation.
- I know that the provided information will be confidential and anonymous.
- I understand that the obtained information will be saved and safeguarded by the researcher in a secure digital platform and in the facilities of the Faculty of Physical Activity Sports Sciences-INEF of the Universidad Politécnica de Madrid.
- I have been informed that my participation does not involve any harm or danger to my physical or mental health.
- I have understood the purpose of the tests and their possible associated risks.
- I have received sufficient oral and written information about the research protocol and have been able to make the necessary questions resolving all my doubts.
- I have been informed that there is no financial compensation for participating in this study.

About the research protocol, I have spoken with:

____

(name of the researcher who provided you information about the study)

I understand that:

- My participation is free, voluntary and anonymous.
- For this study, I have to provide clinical samples: blood and exhaled air (breath sample).
- My diet and exercise habits may change moderately by participating in the study.
- I may refuse to participate or stop participating at any time without giving reasons or receiving any sanction.

POLITÉCNICA

PARTICIPANT CODE: A L

RESEARCHERS: ____________________
(INITIALS OF THE RESEARCHERS)

INEF

I authorize the use of my partial data collected by the research group if I decide to withdraw from the study:

- (1) Yes
- (2) No

I give my consent freely to participate in the study:

- (1) Yes
- (2) No

If you do not agree, describe the reason: ____________________

I wish to know the results of the tests that are done on me:

- (1) Yes
- (2) No

I authorize the use of a part of my anonymized blood sample for further genetic research:

- (1) Yes
- (2) No

I authorize the sample storage of my anonymized blood samples for further studies. Otherwise, the leftover samples will be destroyed.:

- (1) Yes
- (2) No

This document is signed in two copies, leaving one in the possession of each of the parties.

Today's date: ____________________ (DD/MM/YYYY format)

____
Participant's signature

____
Principal researcher's signature

Contact information and principal researchers

Eng MSc Lisset Pantoja- Arévalo: l.pantoja@upm.es
Predoctoral researcher and trainee professor

Dr Eva Gesteiro Alejos: eva.gesteiro@upm.es
Researcher, professor and Co-director of the thesis project

Prof Dr Marcela González-Gross: marcela.gonzalez.gross@upm.es
Principal researcher and Director of the thesis project

Ethics Committee of the Universidad Politécnica de Madrid: secretaria.adjunto.vinvestigacion@upm.es

PARTICIPANT CODE: A L

RESEARCHERS: ______________________
(INITIALS OF THE RESEARCHERS)

END OF THE INFORMED CONSENT FORM

THANK YOU VERY MUCH

HOW TO CITE THIS DOCUMENT:

Pantoja-Arévalo L, Gesteiro E, Pérez-Ruiz M, *et al*. The multifactorial approach and the Food-Allergen Specific Substitutive Diet as a tool to manage and ameliorate adverse reactions to foodstuffs in adulthood: Study Protocol for a Randomized Controlled Trial. The ALASKA study. Appendix 1 Informed Consent Form. *Trials*. 2024.
