# Supplementary material for: The multifactorial approach and the food allergen-specific substitutive diet as a tool to manage and ameliorate adverse reactions to foodstuffs in adulthood: study protocol for a randomized controlled trial—the ALASKA study
Source: Trials. 2024 Jul 20;25:494. doi: 10.1186/s13063-024-08307-2 (PMC11264907; doi:10.1186/s13063-024-08307-2)
Supplement: Supplementary file 4 — Additional file 4: Appendix 2. Inclusion and exclusion criteria form. [file 13063_2024_8307_MOESM4_ESM.pdf]

PARTICIPANT CODE: A L

RESEARCHERS: ______________________

(INITIALS OF THE RESEARCHERS)

APPENDIX 2. INCLUSION AND EXCLUSION CRITERIA FORM

INSTRUCTIONS:
Complete the following Inclusion and Exclusion Criteria Form.

Participant code: ______________________ (If you haven't receive your participant code, please contact the research staff at l.pantoja@upm.es, marcela.gonzalez.gross@upm.es, or gi.imfine@upm.es and request the unique participant code that you will use throughout the entire study as identification. If possible, memorize this code)

Today's date: ______________________ (DD/MM/YYYY format)

Are you participating in another research study, simultaneously?:

- (1) Yes
- (2) No
- (3) I don't know/ I am not sure

Are you smoker?:

- (1) Yes
- (2) No
- (3) I don't know/ I am not sure

If your answer is "Yes", then write down the number of cigarettes which you smoke and choose the frequency (/day, /week, /month, or /year):

Number of cigarettes:

____

Frequency

- (1) per day
- (2) per week
- (3) per month
- (4) per year

Are you taking any type of antibiotics currently?:

- (1) Yes
- (2) No
- (3) I don't know/ I am not sure

You have selected that you are taking some type of antibiotic...
Explain: the name, the concentration, the reason, the medical prescription time and the remaining time to end the antibiotic treatment:

______________________ (e.g. Amoxicillin, 500mg, tooth extraction, 15 days, I have 3 days of treatment left)

Pantoja-Arévalo L, Gesteiro E, Pérez-Ruiz M, *et al*. The multifactorial approach and the Food-Allergen Specific Substitutive Diet as a tool to manage and ameliorate adverse reactions to foodstuffs in adulthood: Study Protocol for a Randomized Controlled Trial. The ALASKA study. Appendix 2 Inclusion and exclusion criteria form. *Trials*. 2024.

PARTICIPANT CODE: A L

RESEARCHERS: ________________________
(INITIALS OF THE RESEARCHERS)

Have you or have you had a Helicobacter pylori bacteria infection (during last year) (with medical diagnosis)?:

- (1) Yes
- (2) No
- (3) I don't know/ I am not sure

Do you have any type of food allergy (with medical diagnosis)?:

- (1) Yes
- (2) No
- (3) I don't know/ I am not sure

Write down the used method or test for your diagnosis of food allergy:

- (1) Skin prick test
- (2) Blood test
- (3) Feces or urine test
- (4) Other

What other method was it used for your diagnosis of food allergy?:

____

Write down the allergens to which you tested positive after taking the food allergy test:

________________________ (e.g. milk, sulfites.)

Do you have any type of food intolerance or food hypersensitivity (with medical diagnosis)?:

- (1) Yes
- (2) No
- (3) I don't know/ I am not sure

Write down the used method or test for your diagnosis of food intolerance or food hypersensitivity:

- (1) Breath test
- (2) Blood test
- (3) Feces or urine test
- (4) Genetic analysis
- (5) Electrodermal test
- (6) Other

What other method was it used for your diagnosis of food intolerance or food hypersensitivity?:

____

Write down the allergens to which you tested positive after taking the food intolerance or food hypersensitivity test:

(e.g. lactose, fructose, glucose, etc.)

Do you have coeliac disease (with medical diagnosis)?:

- (1) Yes
- (2) No
- (3) I don't know/ I am not sure

PARTICIPANT CODE: A L ☐☐☐☐

RESEARCHERS: ________________________

(INITIALS OF THE RESEARCHERS)

Write down the used method or test for your diagnosis of coeliac disease:

- (1) Breath test
- (2) Blood test
- (3) Feces or urine test
- (4) Biopsy
- (5) Other

What other method was it used for your diagnosis of coeliac disease?:

____

Are you taking any type of antidepressant, sleeping pill, or anxiolytic?:

- (1) Yes
- (2) No
- (3) I don't know/ I am not sure

You have selected that you take some type of antidepressant, sleeping pill, or anxiolytic...... Explain: the name, the concentration, the reason, the medical prescription time and the remaining time to end the treatment:

________________________ (e.g. lorazepam, 5mg, for sleep, since 2005 and indefinite prescription)

Do you have a job or lifestyle that may potentially interfere with your regular sleep schedule (e.g. shift night, guards, etc.)?:

- (1) Yes
- (2) No
- (3) I don't know/ I am not sure

You have selected that you have a job or lifestyle that potentially interferes with your regular sleep schedule (e.g. night shift, guards, etc.)...
Explain: the type of work, your weekday work schedule and weekend work schedule:
Type of job:

________________________ (e.g. civil guard, nurse, etc.)

Weekday work schedule:

________________________ (e.g. 11:30 p.m.-6:30 a.m.)

Weekend work schedule:

________________________ (e.g. 9:30 p.m.-4:30 a.m.)

Do you have or have you had some type of eating disorder? (anorexia, bulimia, orthorexia, binge eating, etc.)?:

- (1) Yes
- (2) No
- (3) I don't know/ I am not sure

You have selected that you have had some type of eating disorder (anorexia, bulimia, orthorexia, binge eating, etc.)...
Explain: type of ED, year of diagnosis and current treatment:

________________________ (e.g. bulimia, diagnosed in 2005, discharged)

PARTICIPANT CODE: A L

RESEARCHERS: ______________________
(INITIALS OF THE RESEARCHERS)

Do you have or have you had any type of major surgery that have involved having been hospitalized for one or several days (during the last 5 years)?:

- (1) Yes
- (2) No
- (3) I don't know/ I am not sure

You have selected that you have or have had any type of major surgery that involves hospitalization of one or several days (during the last 5 years)...
Explain: type of major surgery, year of intervention and current treatment/status:

______________________ (e.g. maxillofacial surgery, year 2019, currently fully recovered)

Do you have or have had some type of cancer (during the last 5 years)?:

- (1) Yes
- (2) No
- (3) I don't know/ I am not sure

You have selected that you have or have had some type of cancer (within the last 5 years)...
Explain: type of cancer, year of diagnosis and current treatment:

______________________ (e.g. prostate cancer, diagnosed 2020, cancer remission)

Have you or have you had another illness (during the last 5 years)?:

- (1) Yes
- (2) No
- (3) I don't know/ I am not sure

Write, what other type of illness have you had (during the last 5 years):

____

In general, how many hours of uninterrupted night sleep do you have?:

- (1) 1
- (2) 2
- (3) 3
- (4) 4
- (5) 5
- (6) 6
- (7) 7
- (8) 9
- (9) 9
- (10) 10
- (11) 11
- (12) 12
- (13) more than 12 hours of sleep per night

(number of hours/night)

How many hours of general night sleep do you have?:

- (1) 1
- (2) 2
- (3) 3
- (4) 4
- (5) 5
- (6) 6
- (7) 7
- (8) 9
- (9) 9
- (10) 10
- (11) 11
- (12) 12
- (13) more than 12 hours of sleep per night

(number of hours/night)

AVAILABILITY AND POSSIBILITIES OF PARTICIPANTS

Do you have time availability to attend 2 venous blood sample extractions now and in 5 to 6 months?

- (1) Yes
- (2) No

Do you have the possibility of fasting for at least 8 hours (for blood sample collection purposes)?
Note: If you have any negative recommendations from your doctor related to fasting or blood sample collection procedures, select "No"

- (1) Yes
- (2) No

Are you able to dedicate 30 minutes now and in 5 to 6 months to complete online questionnaires related to this study?

- (1) Yes
- (2) No

PARTICIPANT CODE: A L

RESEARCHERS: ________________________
(INITIALS OF THE RESEARCHERS)

END OF THE INCLUSION AND EXCLUSION CRITERIA FORM

THANK YOU VERY MUCH

HOW TO CITE THIS DOCUMENT:
Pantoja-Arévalo L, Gesteiro E, Pérez-Ruiz M, *et al*. The multifactorial approach and the Food-Allergen Specific Substitutive Diet as a tool to manage and ameliorate adverse reactions to foodstuffs in adulthood: Study Protocol for a Randomized Controlled Trial. The ALASKA study. Appendix 2 Inclusion and exclusion criteria form. *Trials*. 2024.
